# Association between spatial distribution of leukocyte subsets and clinical presentation of head and neck squamous cell carcinoma

**DOI:** 10.3389/fimmu.2023.1240394

**Published:** 2024-01-23

**Authors:** Christoph Netzer, Vanessa von Arps-Aubert, Igor Mačinković, Jens von der Grün, Stefan Küffer, Philipp Ströbel, Andreas von Knethen, Andreas Weigert, Dirk Beutner

**Affiliations:** ^1^ Department of Otorhinolaryngology, Head and Neck Surgery, University Medical Center Göttingen, Göttingen, Germany; ^2^ Institute of Biochemistry I, Faculty of Medicine, Goethe-University Frankfurt, Frankfurt am Main, Germany; ^3^ Department of Radiation Oncology, University Hospital Zurich and University of Zurich, Zurich, Switzerland; ^4^ Department of Radiotherapy and Oncology, University Hospital Frankfurt, Frankfurt, Germany; ^5^ Institute of Pathology, University Medical Center Göttingen, Göttingen, Germany; ^6^ Department of Anesthesiology, Intensive Care Medicine and Pain Therapy, University Hospital Frankfurt, Goethe-University Frankfurt, Frankfurt am Main, Germany

**Keywords:** spatial distribution, leucocyte subsets, head and neck cancer, tumor microenvironment, tumor-associated macrophage, dendritic cell, PD-L1, multiplex immunohistochemistry

## Abstract

**Background:**

Interactions between tumor cells and cells in the microenvironment contribute to tumor development and metastasis. The spatial arrangement of individual cells in relation to each other influences the likelihood of whether and how these cells interact with each other.

**Methods:**

This study investigated the effect of spatial distribution on the function of leukocyte subsets in the microenvironment of human head and neck squamous cell carcinoma (HNSCC) using multiplex immunohistochemistry (IHC). Leukocyte subsets were further classified based on analysis of two previously published HNSCC single-cell RNA datasets and flow cytometry (FC).

**Results:**

IHC revealed distinct distribution patterns of leukocytes differentiated by CD68 and CD163. While CD68hiCD163^lo^ and CD68^hi^CD163^hi^ cells accumulated near tumor sites, CD68^lo^CD163^hi^ cells were more evenly distributed in the tumor stroma. PD-L1^hi^ and PD-1^hi^ cells accumulated predominantly around tumor sites. High cell density of PD-L1^hi^ CD68^hi^CD163^hi^ cells or PD-1^hi^ T cells near the tumor site correlated with improved survival. FC and single cell RNA revealed high variability within the CD68/CD163 subsets. CD68^hi^CD163^lo^ and CD68^hi^CD163^hi^ cells were predominantly macrophages (MΦ), whereas CD68^lo^CD163^hi^ cells appeared to be predominantly dendritic cells (DCs). Differentiation based on CD64, CD80, CD163, and CD206 revealed that TAM in HNSCC occupy a broad spectrum within the classical M1/M2 polarization. Notably, the MΦ subsets expressed predominantly CD206 and little CD80. The opposite was observed in the DC subsets.

**Conclusion:**

The distribution patterns and their distinct interactions via the PD-L1/PD-1 pathway suggest divergent roles of CD68/CD163 subsets in the HNSCC microenvironment. PD-L1/PD-1 interactions appear to occur primarily between specific cell types close to the tumor site. Whether PD-L1/PD-1 interactions have a positive or negative impact on patient survival appears to depend on both the spatial localization and the entity of the interacting cells. Co-expression of other markers, particularly CD80 and CD206, supports the hypothesis that CD68/CD163 IHC subsets have distinct functions. These results highlight the association between spatial leukocyte distribution patterns and the clinical presentation of HNSCC.

## Introduction

Head and neck squamous cell carcinoma (HNSCC) represents the sixth most common tumor entity worldwide (1). In Western regions, tobacco, and alcohol consumption as well as infection with high-risk HPV viruses are major contributors of HNSCC development. Current curative treatment regimens include surgical resection alone, primary radiochemotherapy or tumor resection in combination with adjuvant radio- or radiochemotherapy. Targeted therapies, such as the EGFR inhibitor cetuximab, can be used for chemotherapy-ineligible patients. Immunotherapies, e.g. with PD-1 inhibitors, are FDA approved for cisplatin-refractory recurrent or metastatic (r/m) disease and as first-line therapy for non-resizable or metastatic disease (2–4). A characteristic of HNSCC is their high genetic variability (5–9). Recurrent tumors have a completely altered genetic profile compared to the initial tumors. (10–12). This leaves it uncertain whether specific immunotherapies as a sole therapy can lead to sustained tumor remission. Currently ongoing phase I-III studies on neoadjuvant therapy concepts with immune checkpoint inhibitors will provide new insights (13–15).

HNSCC tumor microenvironment is composed of tumor cells, cells of the surrounding connective tissue, blood and lymphatic vessels, and immune cells (9, 16, 17). Monocytes, macrophages (MΦ) and dendritic cells (DCs) represent critical immune cell types in the tumor microenvironment of HNSCCs. Monocytes and MΦ exhibit great heterogeneity and differ functionally depending on their localization (18–20). They have been shown to influence tumor development by promoting fibrosis and angiogenesis, suppressing T-cell responses through cytokines, (cross-)presenting tumor antigens together with co-inhibitory molecules such as PD-L1, and degrading key metabolites required for T-cell proliferation such as L-arginine. Moreover, their localization in the tumor microenvironment appears to be an influential prognostic parameter (21, 22). Like MΦ/monocytes, DCs exhibit great heterogeneity within HNSCCs and can exert both tumor activating and inhibitory effects (23–25). Their cell density in HNSCC seems to correspond to HPV status as well as T-cell infiltration, among other factors (23, 24).

Given their great heterogeneity, the study of tumors, in addition to being a scientific challenge, became at the same time a major technical and financial challenge. Technologies such as single cell sequencing or flow cytometry now allow highly targeted and differentiated typing cells in the tumor microenvironment. However, these techniques do not reflect spatial information of these cell types. Especially due to the intratumoral heterogeneity, spatial differentiation of the microenvironment allows for a much more sophisticated and precise assessment of tumors (26, 27). Immunohistochemical (IHC) methods provide spatial information of the visualized cells within the tumor microenvironment. Currently, there is a rapid development of immunohistochemical techniques that allow phenotyping of single cells and simultaneous investigation of their spatial relationships (26, 28). One of them is based on cyclic immunofluorescence staining of multiple epitopes with tyramide dyes (29). The epitope-specific antibodies are removed after each staining cycle, but the dyes remain covalently bound to their epitopes. This allows simultaneous visualization of multiple epitopes in one section, with the origin of the primary antibody host playing only a minor role for the staining.

The focus of this study was to evaluate the relationships between the spatial localization and function of cell subsets distinguishable by CD68 and CD163 and their functional roles in the HNSCC tumor microenvironment. Both proteins are expressed in particular by monocytes, MΦ, and DCs. CD68 is mainly localized lysosomally and endosomally (30). It is highly expressed by MΦ, but also by DCs and other cells such as neutrophils and by some tumor cells. In MΦ it is associated with a pro-inflammatory M1-like phenotype. The predictive value of CD68 as a prognostic marker of survival in cancer patients is highly controversial (30–32). The localization of CD68^+^ MΦ in the tumor microenvironment, among other factors, appears to play a major role for clinical outcome (32). CD163 is almost exclusively expressed in monocytes, MΦ and DCs (33, 34). In MΦ it is associated with an M2-like polarization. High expression in tumors is correlated with poor prognosis in different cancers, among others in HNSCC (31, 33, 35). To date, few histological studies have investigated the co-expression of these two markers, which allows more accurate conclusions to be drawn about their simultaneous expression on a single cell as well as their spatial distribution in the tumor. In this regard, we have developed several antibody panels for tyramide-based multiplex IHC, to investigate the spatial relationships of CD68/CD163 subsets relative to other cells in the microenvironment of human HNSCCs. CD68/CD163 subsets were further classified based on analysis of two previously published HNSCC single-cell RNA datasets and by flow cytometry (FC), distinguishing between monocyte, MΦ, and dendritic cell subsets (DCs).

## Material and methods

### Patient cohorts

The immunohistochemical examinations were performed on head and neck squamous cell carcinomas (HNSCC), CIS and tumor-free controls, which were taken during regular procedures and were no longer required for routine diagnostics. HPV- and non-HPV-associated oropharyngeal carcinomas of T stages T1-2, N stages N0-N2 and M stages M0 were used for the investigations on the whole sections. The control group consisted of tonsils from non-tumor patients who had undergone tonsillectomy due to recurrent acute tonsillitis. The tissue mircroarray (TMA) set 1 is composed of HNSCCs from different regions. Here, HNSCC, CIS and corresponding tumor-free tissues were taken from HNSCC patients. TMA set 2, analogous to the whole section, consists of HPV- and non-HPV-associated oropharyngeal carcinomas and tonsils of tumor-free donors as a control group. The suitability of all samples was validated by a pathologist. For the flow cytometric examinations, native tissue from histologically confirmed oropharyngeal carcinomas was taken during regular tumor resections. The exact composition and clinicopathological parameters are shown in **Supplementary Tables 1**-**3**. All tissue samples in the study were pre-selected by a pathologist. Written informed consent was obtained from all patients and the study was approved by the ethics committees of the University Medical Center Göttingen (ethical vote 25/7/18), the institutional review board of the University Cancer Center (UCT) and the ethical committee at the University Hospital Frankfurt (project numbers: 4/09, SKH-1-2019). With regard to the single-cell mRNA datasets from Cillo et al. (GSE139324 (9)) and Kürten et al. (GSE164690 (17)) the investigators provided written informed consent for all human patient samples prior to donation. The studies were approved by the review board of the University of Pittsburgh Cancer Institute (protocol 99-069). 10x genomics dataset of PBMCs (Published on September 14th, 2021) is licensed under the Creative Commons Attribution license. 

### Multiplexed immune fluorescence staining on FFPE tissues

For the immunohistochemical stainings (IHC), 4 µm sections of Formalin-Fixed Paraffin-Embedded (FFPE) tissue were prepared. To ensure optimal tissue adhesion, the slides were heated 60°C for 30 minutes at in a drying oven. Subsequently, the slides were deparaffinized in xylene and a descending alcohol series. Prior to sequential staining, antibodies were validated and optimized by single stainings, followed by determination of the optimal staining and fluorophore sequence for multiplexed fluorescence imaging. Sequential multiplexed fluorescence staining was performed using a BOND-RX Multiplex IHC Stainer (Leica Biosystems). Slides were deparaffinised and then sequential stained for each epitope with Opal fluorophores (FP1500001KT, FP1487001KT, FP1494001KT, FP1488001KT, FP1495001KT, FP1496001KT, FP1497001KT, FP1501001KT, Akoya Biosciences) as follows: Antigen retrieval was performed at pH6 or 9 using BOND Epitope Retrieval Solution 1 and 2 (#AR9961, #AR9640, Leica Biosystems) for 20 min at 95°C followed by three washing steps with BondTM Wash Solution (#AR9590, Leica Biosystems). To reduce endogenous peroxidase activity, treatment with 3% H_2_O_2_ was exclusively carried out for 10 min before the first antigen staining. After three washing steps and treatment with Antibody Diluent/Block (#ARD1001EA, Akoya Biosciences) for 5 min to reduce non-specific antibody binding, incubation with primary antibody was performed for 30 min at room temperature, followed by three washing steps. Slides were then incubated with the secondary antibody for 10 min at room temperature, followed by three washing steps and staining with Opal fluorophores (Akoya Biosciences) for 10 min. Slides were washed 3 times before antigen retrieval for the next primary antibody. The previous steps were repeated for each antigen. In the final staining with Opal-780, slides were incubated with TSA-DIG instead of Opal fluorophore followed by 2 x incubation with Opal 780 for 10 and 60 min and 3 washing steps. Finally, nuclei were stained with Spectral DAPI (#FP1490, Akoya Biosciences), followed by 3 washing steps and covering with Fluoromount™ Aqueous Mounting Medium (#F4680, Sigma Aldrich) and cover slides (#631-0158, VWR). The antibody and fluorochrome dilutions as well as the exact staining protocol are described in **Supplementary Tables 4**-**7**.

### Fluorescence imaging

Multiplex imaging was performed in a Vectra Polaris Automated Quantitative Pathology Imaging System (Akoya Biosciences) at 20x magnification. On whole sections, 7-fold multispectral imaging was performed on 10-15 randomly selected representative regions of 3 x 3 mm size, which had a tumor or squamous epithelium to stroma ratio of 1:1. For tissue microarrays, 7-fold multispectral imaging was performed, with each TMA of approximately 1.2 mm in diameter being fully detected.

### Machine learning-based image analysis and processing

Image analysis and processing was performed using the automated image analysis software inForm (Akoya Biosciences) which comprises several consecutive processing steps as listed below. Fluorescence images obtained on the Vectra Polaris Automated Quantitative Pathology Imaging System were first compensated for individual fluorophore spectral overlap and autofluorescence (Spectral unmixing). For whole slides, compensation was based on individual marker staining and unstained samples, for TMAs using synthetic libraries (MOTiF scan). By repeated training on representative samples, the software was trained on the basis of the Random Forest machine learning algorithm to distinguish independently between squamous epithelium/head and neck squamous cell carcinoma and stroma (Tissue segmentation). By means of computer vision techniques utilizing DAPI nuclear and immunohistochemical stainings, cells were subsequently segmented into nucleus, cytoplasm and membrane (Cell segmentation). The software was then trained to differentiate between predefined cell subsets on the basis of a multinomial logistic regression model (Phenotyping). On whole slides, distinction was made between 1. CD68^hi^ and/or CD163^hi^ cells, 2. CD45^hi^ (CD68^lo^CD163^lo^) cells (leukocytes), 3. CK^hi^ cells (keratinocytes or tumor cells). In the TMAs, the CD68/CD163 subsets were already differentiated in inForm via “phenotyping”. This resulted in the following cell subsets: 1. CD68^hi^CD163^lo^, 2. CD68^hi^CD163^hi^, 3. CD68^lo^CD163^hi^, CD3^+^ cells (T cells) and 5. CK^hi^ cells (keratinocytes or tumor cells). After satisfactory independent tissue and cell segmentation and phenotyping of the training samples, the specificity of recognition was randomly checked on test samples and image processing was finally performed on all test samples. More details on the inForm software can be obtained at https://www.AkoyaBiosciencesbio.com.

### R-based image and statistical analysis

Staining quality was assessed together with a trained pathologist on each individual spectrally unmixed image before further processing. Analysis of the images and data processed by inForm software was carried out in R programming language using the integrated development environment RStudio (open-source licensed software, https://posit.co/download/rstudio-desktop/), including the phenoptr package (Akoya Biosciences, https://akoyabio.github.io/phenoptr/). inForm provides, among other things, a confidence value for each of the predefined phenotypes. Only cells with a confidence level of more than 50% were considered for further processing and statistical analysis. For whole slides, further processing initially involved subdividing the CD68^hi^ and/or CD163^hi^ cells into CD68^hi^CD163^lo^, CD68^hi^CD163^lo^ and CD68^lo^CD163^hi^ cells based on the fluorophore thresholds for CD68 and CD163. The threshold values are based on the normal distribution of the respective expression values. In the TMAs, the determination of PD-L1^hi/lo^ and PD-1^hi/lo^ cells was also carried out on the individual fluorophore thresholds. Further image and statistical analysis utilized the following elements of the phenoptr package: density_at_distance(), find_nearest_distance(), count_touching_cells(). Links to the corresponding description of the functions mentioned are listed in the **supplementary material**. Following R-based image analysis and data processing, Prism (GraphPad software) was used to perform correlation analyses on clinical parameters.

### Data analysis of single cell NGS data

Data analysis was carried out following the Seurat (v. 4.3.0) pipeline (https://satijalab.org/seurat/index.html) in R (4.2.2) using RStudio (2022.12.0 Build 353) IDE. In brief, data sets (GSE139324 (9), GSE164690 (17), and pbmc10k (Published on September 14th, 2021. This dataset is licensed under the Creative Commons Attribution license)) were imported using Read10X/CreateSeuratObject function and corresponding cohorts were merged. After inspection, cells with less than 200 and more than 3,000 (GSE139324 and pbmc10k datasets) or 5,000 (GSE164690 dataset) Feature RNA, and more than 10% (GSE139324 and GSE164690 datasets) or 15% (pbmc3k dataset) mitochondria RNA were filtered out from the analysis, following the scRNA-seq quality control guidelines (36, 37). The remaining cells were normalized using the SCTransform function, using percent mitochondrial reads as a covariate for regression (38). Before clustering, cells with the following characteristics were subsets: CD14^+^ or ITGAM^+^, APOE^+^ or MRC1^+^, CD68^+^ or CD163^+^, MPO^-^ or IL3RA^-^ or CD1C^-^. Subset cells were clustered using the RunUMAP function (37), and differentially expressed genes between clusters were identified using the FindAllMarkers (39, 40) function.

### Preparation of tumor single-cell suspensions

Tumors were minced with a scalpel into approx. 2 x 2 x 2 mm pieces and transferred to an enzymatic digestion solution in RPMI (#130-095-929, Miltenyi). Using the gentleMACS Dissociator (Miltenyi Biotec), mechanical dissociation was carried out, followed by enzymatic digestion at 37° C for 15 minutes in a shaking incubator and renewed mechanical dissocation using the gentleMACS Dissociator. The cell suspensions were then passed through a 100 µm and 40 µm cell strainer (#352360, #352340, Corning), diluted with MACS buffer, and centrifuged at 300 x g for 5 min (4° C). The supernatant was discarded, cells were washed again with MACS buffer and the centrifugation step was repeated.

### Macrophage generation

Human macrophages (MΦ) were differentiated from buffy coat-derived monocytes. PBMCs were separated from buffy coats using Ficoll gradient centrifugation (35 min, 440 x g, RT, brake to 2), and were washed twice with PBS (centrifugation: 5 min, 500 x g, RT, maximum brake). PBMCs were subsequently taken up in MACS buffer and monocytes were positively selected using CD14 MicroBeads (#130-050-201, Miltenyi Biotec) according to the manufacturer’s instructions. CD14^+^ cells were then incubated for 7 d in macrophage-SFM (1X) (Gibco™, #12065074) + 100 ng/ml MCSF without further additives (M0 MΦ) or additionally from day 3 with 50 ng/ml IFNγ and 10 ng/ml LPS (M1) or 10 ng/ml IL4 (M2 MΦ) (6-well plates, 2 x 10^6^ cells, 2 ml medium, 37°C, 20% O_2_, 7.5% CO_2_).

### Flow cytometry

Single-cell suspensions were incubated with Fc receptor blocking solution (#422301, BioLegend) for 20 min on ice followed by extracellular staining with fluorochrome-conjugated antibodies. After 2 washing steps with PBS, and fixation with 4% neutral buffered formalin for 10 min at RT followed by 2 washing steps, cells were permeabilized twice for 10 min at RT with 0,5% saponin in MACS buffer. Intracellular staining was then carried out for 20 min at RT followed by 2 washing steps. The following antibodies were used for analysis of human HNSCCs: Anti-CD1c-BB515 (#565054, BD Horizon), Anti-CD3-APC (#300312, BioLegend), Anti-CD14-PerCP (#325632, BioLegend), Anti-CD33-APC-Cy7 (#366614, BioLegend), Anti-CD45-BV605 (#304042, BioLegend), Anti-CD45-BV605 (#304042, BioLegend), Anti-CD64-PE (#305008, BioLegend), Anti-CD68-PE-CF594 (#564944, BD Horizon), Anti-CD80-BV785 (#305238, BioLegend), Anti-CD163-BV421 (#333612, BioLegend), Anti-CD206-PE-Cy5 (#321108, BioLegend), anti-CD274-PE-Cy7 (#558017, BD Pharmingen), anti-HLA-DR-AF700 (#307626, BioLegend), anti-MerTK-BV711 (#367620, BioLegend). Extracellular and intracellular CD163 staining on MDMs was performed using the following antibodies: Anti-CD68-PE-CF594 (#564944, BD Horizon), Anti-CD163-BV421 (#333612, BioLegend), Anti-CD163-BUV496 (#750581, BD OptiBuild), Anti-HLA-DR-AF700 (#307626, BioLegend), Anti-MerTK-BV711 (#367620, BioLegend). Since only < 10% apoptotic or dead cells with negligible effects on the binding specificity of the antibodies used were detected in random tumor sample controls with live/dead staining, live/dead staining was not taken into account in the measurements listed. Flow cytometric analysis was performed using an LSRII/Fortessa flow cytometer. Data analysis was carried out by FlowJo V10.8.1 (TreeStar). All antibodies and secondary reagents were titrated to determine optimal concentrations. Comp Beads (#424602, BioLegend), human PBMCs or monocyte-derived MΦ (MDMs) were used to generate single color compensation samples to create multicolor compensation matrices and to evaluate multicolor panels. Fluorescence minus one controls and populations with known negativity for specific antigens were used to validate gating. Instrument calibration was checked daily using Cytometer Setup and Tracking Beads (#642412, BD Biosciences).

### Statistics

Unless otherwise indicated, data are presented as mean ± standard error of the mean. Statistically significant differences between groups were determined using the Wilcoxon rank sum test with Bonferroni correction. Significance levels between groups are indicated as asterisks above the graphs. * p<0.05, ** p<0.01, *** p<0.001, **** p<0.0001.

## Results

### Different spatial distribution of MΦ markers CD68 and CD163 in the HNSCC microenvironment

**Figure 1:**
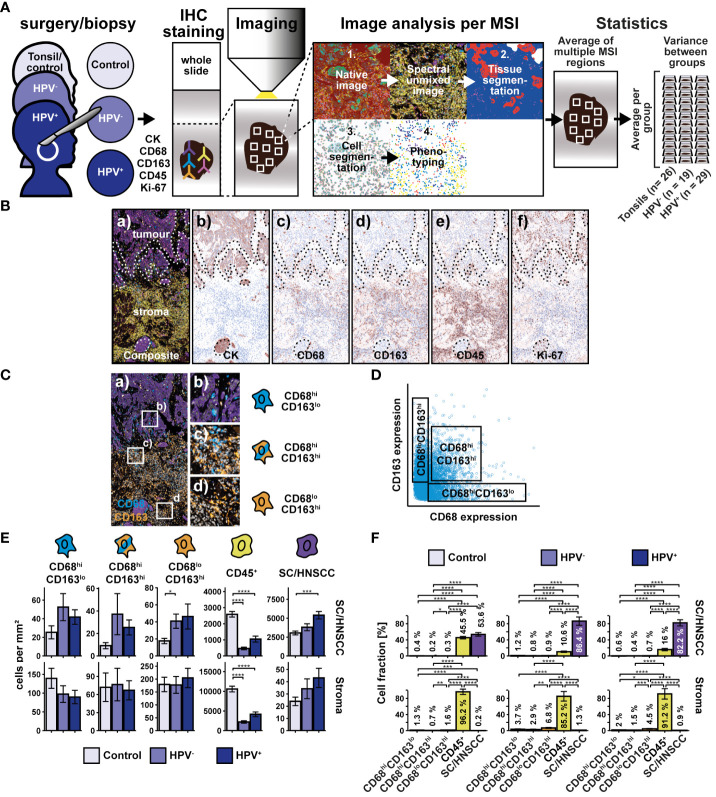
Workflow and cohort overview for multiplex immunohistochemical examination of whole slides of tonsils, non-HPV-associated and HPV-associated HNSCCs. **(A)** Sequential fluorescence staining was performed for cytokeratin (CK), CD68, CD163, CD45, and Ki-67 followed by fluorescence imaging of multiple regions (MSI) per slide. Image analysis and processing was then carried out by using the inForm software (Akoya Biosciences) and comprised the followingsteps: 1. “Spectral unmixing” of the image into its stain components, 2. “Tissue segmentation” of differentiated tissue regions (SC/HNSCC and stroma), 3. “Cell segmentation” into membrane, cytoplasm and nucleus, 4. “Phenotyping” to identify cell types specified by the investigator. During phenotyping, distinction was made between the following subsets: CD68^hi^ and/or CD163^hi^ cells, leukocytes (CD45^+^CD68^lo^CD163^lo^) and keratinocytes or tumor cells (CK^hi^). After image analysis and processing, a visual quality control of each sample was performed. 26 Tonsils (controls) from patients with recurrent acute tonsillitis, 19 non-HPV-associated (HPV^-^) and 29 HPV-associated (HPV^+^) oropharyngeal carcinomas were included in the statistical analysis (for details, see **Supplementary Table 1**). Further image and statistical analysis was carried out in R, where initially the CD68^hi^ and/or CD163^hi^ subset was further subdivided into CD68^hi^CD163^lo^, CD68^hi^CD163^lo^ and CD68^lo^CD163^hi^ cells based on the threshold values for CD68 and CD163. **(B)** Composite image (a) highlighting tumor and stromal regions and individual staining for CK (b), CD68 (c), CD163 (d), CD45 (e), and Ki-67 (f). The boundary between squamous epithelium (SC) or HNSCC and stroma (ST) (SC/HNSCC-ST boundary) has been highlighted here as a dashed line. **(C)** Composite image of CD68^hi^CD163^lo^ cells (a), CD68^hi^CD163^hi^ cells (b) and CD68^lo^CD163^hi^ cells (c). **(D)** CD68 vs CD163 dot plot highlighting CD68/CD163 subsets determined in R on the via threshold. **(E)** Quantification of cell densities (per mm2) of the above CD68/CD163 subsets, leukocytes (CD45^+^), and SC/HNSCC in tumor nests (HNSCC) or plate epithelium (controls) and stroma. **(F)** Same samples as in E but summarized by tissues and plot of relative cell densities (%). The significance levels (by Wilcoxon rank sum test) between the groups are indicated as asterisks above the plots. *p<0.05, **p<0.01, ***p<0.001, ****p<0.0001.

To investigate the distribution of the MΦ markers CD68 and CD163 within the tumor microenvironment of head and neck squamous cell carcinoma (HNSCC), whole slides of HPV-associated, non-HPV-associated oropharyngeal cancer and tonsils as tumor-free controls were sequentially stained using antibodies against CD68, CD163, CK, CD45 and Ki-67 (**Figure 1A**). Initially, CD68 and CD163 expression were analyzed (**Figure 1B**). CD68 was found predominantly in and around pan-cytokeratin positive (CK^+^) tumor nests, whereas CD163 was expressed more consistently, throughout the tumor stroma.

### Spatial distribution of CD68/CD163 cell subsets and CD45^+^ leukocytes in HNSCC

We next statistically validated the previous observations. For this, we determined thresholds for both markers based on the averaged expression values, which we used to divide the CD68/CD163 subgroups into CD68^hi^CD163^lo^, CD68^hi^CD163^hi^, and CD68^lo^CD163^hi^ (**Figures 1C, D**). Since CD68 and CD163 are expressed not only by MΦ but also by other cells, we do not refer to CD68/CD163-expressing cells as MΦ at this point. The cell density of the thus defined CD68/CD163 subsets, CD45^+^ leukocytes and keratinocytes or HNSCC (CK^+^) in squamous epithelium or tumor nests and stroma was then determined (**Figures 1E, F**). In HNSCC, we observed significantly lower CD45^+^ leukocyte density compared with controls (tonsils) (**Figure 1E**). This affected both stroma and tumor nests compared to squamous epithelium. Significant differences between HPV- and non-HPV-associated tumors were not detected. Within the CD68/CD163 subsets, we found a higher cell density in the CD68^lo^CD163^hi^ subset in non-HPV-associated tumors compared with the control group. Beyond that, no significant differences were found, although trends emerged. The cell density of CD45^+^ leukocytes and CD68/CD163 subsets was generally higher in the stroma than in tumor nests (HNSCC) or squamous epithelium (controls). Within all leukocytes (CD45^+^ leukocytes and CD68/CD163 subsets combined), CD45^+^ leukocytes generally had the highest cell density relative to total cell density (16.0 - 45.5% in tumor nests or squamous epithelium and 85.2 - 96.2% in stroma) (**Figure 1F**). The cell density of CD68/CD163 subsets varied between 0.2 and 0.7% (tumor nests and squamous epithelium, respectively) and 0.2 - 6.8% (stroma).

**Figure 2:**
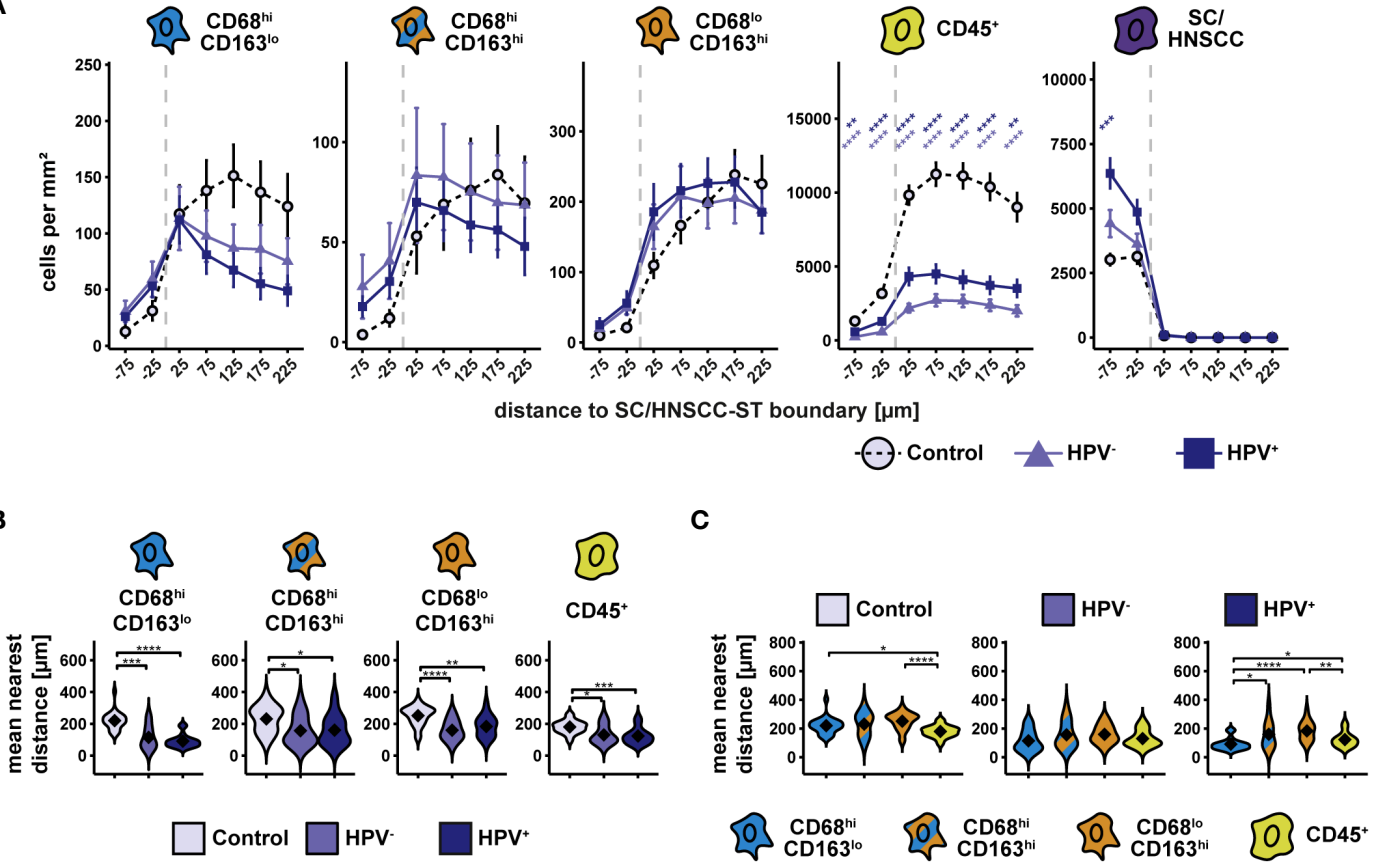
Spatial analysis of CD68/CD163 subsets, leukocytes and SC/HNSCC in whole slides of tonsils, non-HPV-associated and HPV-associated HNSCCs. **(A)** Cell density as a function of distance to squamous epithelium (SC) or HNSCC-stroma (ST) boundary (SC/HNSCC-ST boundary). The distance to the SC/HNSCC-ST boundary is plotted on the X-axis. The vertical dashed line indicates the border between the stroma and the tumor or squamous epithelium. Negative X values reflect distances to SC/HNSCC-ST boundary within the tumors or squamous epithelium, positive X values distances in the adjacent stroma. Each line represents a tissue type (control (tonsil) = dashed black, non-HPV-associated (HPV^-^) tumors = light blue or HPV^-^associated (HPV^+^) tumors = dark blue), each point represents the mean value of multiple samples. The error bars represent the standard error. The significance levels (by Wilcoxon rank sum test) between the groups are color-coded and indicated vertically as asterisks above the mean values. *p<0.05, **p<0.01, ***p<0.001, ****p<0.0001. **(B)** and **(C)** Nearest distance of cell types to tumor cell or keratinocyte. Each violin plot reflects the averaged nearest distances of CD68/CD163 subsets (CD68^hi^CD163^lo^, CD68^hi^CD163^hi^ and CD68^lo^CD163^hi^) and leukocytes (CD45^+^) to next tumor cell or keratinocyte of the individual samples subdivided by tissue type (control (tonsil), HPV^-^ and HPV^+^ HNSCC). For better illustration, these have been sub sorted and color-coded by tissue type **(B)** or cell type **(C)**. The Y-axis reflects the distance to the nearest tumor cell or keratinocyte. The mean values are shown as diamonds. The Wilcoxon rank sum test was used to determine the level of significance of the differences between the two groups. The significance (by Wilcoxon rank sum test) levels between the groups are indicated as asterisks above the plots. *p<0.05, **p<0.01, ***p<0.001, ****p<0.0001.

To include spatial information, we determined the cell density of the three CD68/CD163 subsets and the other CD45^+^ leukocytes as a function of distance to squamous epithelium (SC) or HNSCC-stroma (ST) boundary (SC/HNSCC-ST boundary) (**Figure 2A**). Particularly in HNSCC, the cell density of CD68^hi^CD163^lo^, but also of CD68^hi^CD163^hi^ subset was increased in and in the immediate vicinity of the tumor nests, whereas in the control group it was generally high in the stroma and decreased toward squamous epithelium. In contrast, the cell density of CD68^lo^CD163^hi^ subset was increased almost exclusively in the stroma around the tumors or the squamous epithelium, respectively. Differences between HNSCC and controls in this CD68/CD163 subset were not detected. Within the CD45^+^ leukocytes, the cell density was generally lower in the entire tumor tissue compared to the tonsils. In all three groups, the leukocyte count was higher in the stroma than in the tumor nests or squamous epithelium. The mean nearest distance (MND) between CD68/CD163 subsets and T cells to tumor cells in HNSCC or keratinocytes in control tissue was determined as another distance and density correlate (**Figures 2B, C**). Within all CD68/CD163 subsets as well as of the CD45^+^ leukocytes the MND to next tumor cells or keratinocytes was significantly lower in the HNSCC groups compared to the controls (**Figure 2B**). The strongest effect size was observed within the CD68^hi^CD163^lo^ subset. It was further noticed that the MND varied between the CD68/CD163 subsets in the controls and HPV-associated tumors but not in non-HPV-associated tumors (**Figure 2C**). Thus, there were clear spatial distribution differences of the CD68/CD163 subsets as well as the other CD45^+^ leukocytes within the HNSCC and in the comparison between HNSCC and tonsils. CD68^hi^CD163^lo^ and CD68^hi^CD163^hi^ cells as well as T cells appear to be concentrated primarily at the SC/HNSCC-ST boundary and in tumor nests, while CD68^lo^CD163^hi^ cells are rather ubiquitously distributed in the stroma.

### Spatial distribution of PD-L1 expressing cells in HNSCC

**Figure 3:**
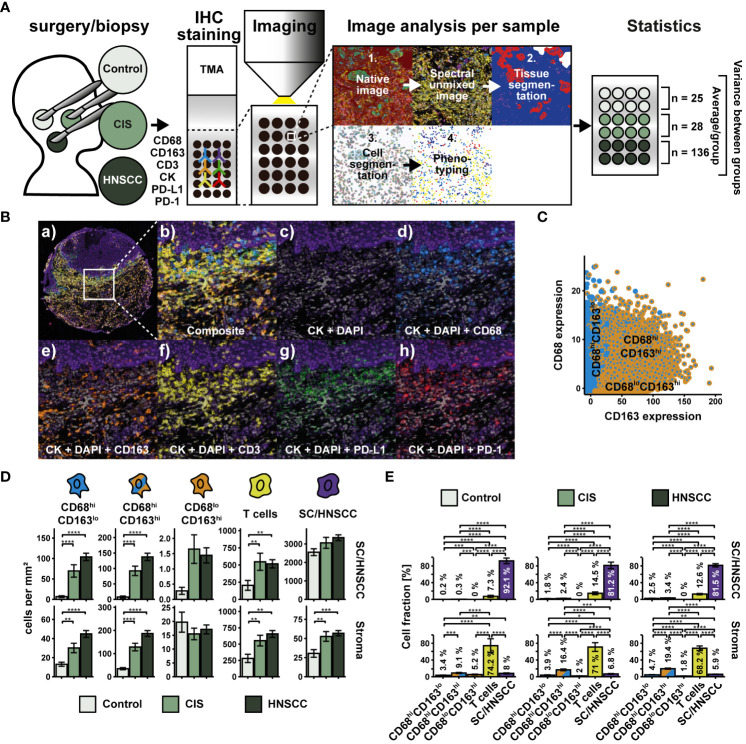
Workflow and cohort overview for multiplex immunohistochemical examination of TMAs. **(A)** TMAs were composed of 27 tumor-free samples (controls), 39 CIS and 145 carcinomas (HNSCC) from HNSCC of different regions (for details, see **Supplementary Table 2**). Sequential fluorescence staining was performed for cytokeratin (CK), CD68, CD163, CD3, PD-L1 and PD-1 followed by fluorescence imaging. Image analysis and processing was then carried out by using the inForm software (AKOYA BIOSCIENCES) according to **Figure 1** with the following modifications: During phenotyping, a distinction was made between CD68^hi^CD163^lo^ cells, CD68^hi^CD163^lo^ cells, CD68^lo^CD163^hi^ cells, T cells (CD3^+^) and keratinocytes or tumor cells (CK^hi^). After image analysis and processing, a visual quality control of each sample was performed. 25 controls, 28 CIS and 136 HNSCCs were included in the statistical analysis. Further image and statistical analysis was carried out in R. **(B)** Composite image (a) and (b), double staining for DAPI and CK (c) and triple staining for DAPI, CK and CD68 (d), CD163 (e), CD3 (f), PD-L1 (g) or PD-1 (h). **(C)** Dot plot visualizing CD68 and CD163 expression within CD68/CD163 subsets defined by deep learning. **(D)** Quantification of cell densities (per mm^2^) of the above CD68/CD163 subsets, T cells (CD3^+^), and SC/HNSCC in tumor nests (HNSCC) or plate epithelium (controls) and stroma. **(E)** Same samples as in **(D)** but summarized by tissues and plot of relative cell densities (%). The significance levels (by Wilcoxon rank sum test) between the groups are indicated as asterisks above the plots. *p<0.05, **p<0.01, ***p<0.001, ****p<0.0001.

We wondered what other characteristics distinguished the three CD68/CD163 subsets from each other. In a study on HNSCC, flow cytometry (FC) showed that MΦ had the highest PD-L1 surface expression compared to other cell types in the tumor microenvironment (17). These data were supported by multiplex IHC data in which MΦ (defined as CD68^+^) expressed PD-L1 particularly at the SC/HNSCC-ST boundary and in tumor tissue. In addition, there was an increased T-cell infiltrate at the tumor border and within the tumor nests. However, MΦ subsets were not investigated. We therefore asked if PD-L1 expression in HNSCC varied between the spatially separated CD68/CD163 subsets we identified before. Moreover, we were interested in the extent to which cell-cell contacts occur between the PD-L1-expressing CD68/CD163 subsets and PD-1-expressing T cells. TMAs from HNSCC, CIS, and tumor-free samples were therefore stained sequentially with antibodies against CD68, CD163, CK, CD3, PD-1, and PD-L1 (**Figures 3A, B**). In contrast to whole slides, the TMA consisted of HNSCCs from different regions and corresponding CIS and tumor-free mucosa (controls) obtained from the same HNSCC patients. The classification of the three CD68/CD163 subgroups in this case was performed as part of a deep learning-based phenotype determination (**Figure 3C**, see Material and Methods section for details). We observed higher cell density of CD68^hi^CD163^lo^, CD68hiCD163^hi^ cells, and T cells in HNSCC and CIS compared with controls (**Figure 3D**). This affected both stroma and tumor nests compared with squamous epithelium. Leukocyte density was generally higher in the stroma than in the tumor nests (HNSCC) or squamous epithelium (controls and CIS). Within leukocytes, T cells generally exhibited the highest cell density relative to total cell density (7.3 - 14.5% in tumor nests or squamous epithelium and 68.2 - 74.2% in stroma), followed by CD68^hi^CD163^hi^ cells (0.3 - 3.4% in tumor nests or squamous epithelium and 9.1 - 19.4% in stroma), and CD68^hi^CD163^lo^ cells (0.2 - 2.5% in tumor nests or squamous epithelium and 3.4 - 4.7% in stroma) (**Figure 3E**). Next, we validated cell densities overall and as a function of distance from the SC/HNSCC-ST boundary in TMA based on whole slides and another TMA dataset of oropharyngeal carcinomas (**Supplementary Figures 1**, **2**), in both cases utilizing tonsils from tumor-free patients as control tissue. Of particular interest were systematic differences due to control tissue type and different tumor HNSCC loci. Non-HPV and HPV-associated tumors of whole slides (WS-OPX) and oropharyngeal carcinoma TMAs (TMA-OPX) were combined for comparison with HNSCC TMAs (**Supplementary Figures 1A–D**). Within the HNSCC-TMAs (TMA-HNSCC), we additionally distinguished the oropharyngeal carcinomas (HNSCC : OPX) to account for a possible influence of tumor location on cell density (**Supplementary Figures 1E, F**). We found that the whole slide and oropharyngeal carcinoma TMA controls exhibited significantly higher leukocyte density than the HNSCC TMAs (**Supplementary Figure 2A**), which suggests an influence of the nature of the control tissue. This difference was particularly pronounced in the stroma. Within the HNSCC tissues (**Supplementary Figure 2B**), cell densities of CD68^hi^CD163^lo^ cells in tumor nests or squamous epithelium were lower in whole slides and oropharyngeal carcinoma TMAs than in HNSCC TMAs. By contrast, CD68^hi^CD163^lo^ density in stroma was higher in whole slides than in oropharyngeal carcinoma and HNSCC TMAs. The density of CD68^hi^CD163^hi^ cells was generally lower in the whole slides and oropharyngeal carcinoma TMAs than in the HNSCC TMAs, both in tumor nests or squamous epithelium and in the stroma. Conversely, the density of CD68^lo^CD163^hi^ cells in the whole slides and TMAs of oropharyngeal carcinomas was significantly higher than in the HNSCC TMAs in both tumor nests or squamous epithelium and stroma. CD45^+^ cell/T-cell density in tumor nests or squamous epithelium only differed between whole slides and the oropharyngeal carcinomas of HNSCC TMAs. The density within the TMAs was slightly lower. By contrast, it was clearly highest in the stroma of the whole slides, followed by the oropharyngeal carcinoma TMAs. Considering the spatial cell location (**Figures 2C, D**), it becomes apparent that the density differences between whole slides, oropharyngeal carcinoma TMAs and HNSCC TMAs were particularly pronounced at greater distances from the SC/HNSCC-ST boundary (**Figures 2C, D**). In HNSCC tissues, this mainly affected CD68^lo^CD163^hi^ cells and CD45^+^/T cells. Visual comparison of the slides confirmed these observations. HNSCC-TMAs exhibited a significantly higher tumor-to-stroma ratio compared to whole slides and oropharyngeal TMAs, which explained the density differences especially at larger distances to SC/HNSCC-ST border. Generally, no significant density differences were found between the selected oropharyngeal carcinomas within HNSCC TMAs and the HNSCC TMAs as a whole. Thus, tumor entity does not appear to have a significant influence here. Despite certain methodological differences, we considered the comparability between TMAs and whole slides to be appropriate.

**Figure 4:**
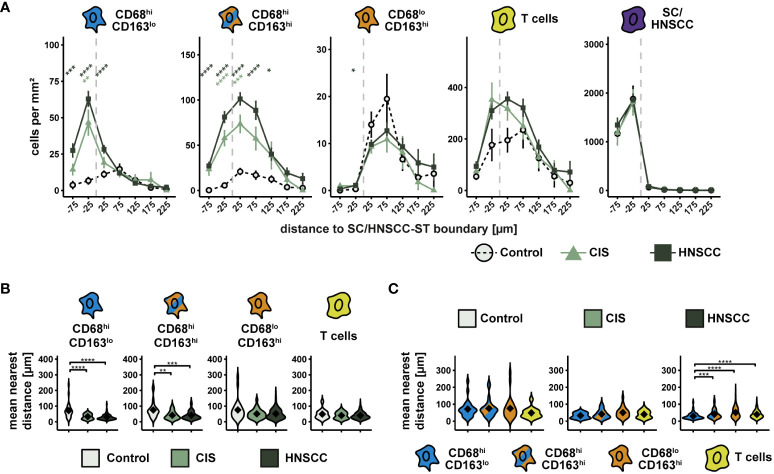
Spatial analysis of CD68/CD163 subsets, T cells and SC/HNSCC in TMAs of controls, CIS and HNSCC. **(A)** Cell density as a function of distance to squamous epithelium (SC) or HNSCC-stroma (ST) boundary (SC/HNSCC-ST boundary). The distance to the SC/HNSCC-ST boundary is plotted on the X-axis. The vertical dashed line indicates the border between the stroma and the tumor or squamous epithelium. Negative X values reflect distances to SC/HNSCC-ST boundary within the tumors or squamous epithelium, positive X values distances in the adjacent stroma. Each line represents a tissue type (control = dashed black, CIS = light green or HNSCC = dark green), each point represents the mean value of multiple samples. The error bars represent the standard error. The significance levels (by Wilcoxon rank sum test) between the groups are color-coded and indicated vertically as asterisks above the mean values. *p<0.05, **p<0.01, ***p<0.001, ****p<0.0001. **(B, C)** Nearest distance of cell types to tumor cell or keratinocyte. Each violin plot reflects the averaged nearest distances of CD68/CD163 subsets (CD68^hi^CD163^lo^, CD68^hi^CD163^hi^ and CD68^lo^CD163^hi^) and T cells (CD3^+^) to next tumor cell or keratinocyte of the individual samples subdivided by tissue type (control, CIS and HNSCC). For better illustration, these have been sub sorted and color-coded by tissue type **(B)** or cell type **(C)**. The Y-axis reflects the distance to the nearest tumor cell or keratinocyte. The mean values are shown as diamonds. The significance (by Wilcoxon rank sum test) levels between the groups are indicated as asterisks above the plots. *p<0.05, **p<0.01, ***p<0.001, ****p<0.0001.

Compared with controls, increased cell density of CD68^hi^CD163^lo^ cells, CD68^hi^CD163^hi^ cells, and T cells was observed in and around HNSCC/CIS cell nests (**Figure 4A**). In contrast, the cell density of CD68^lo^CD163^hi^ cells remained relatively uniform in the stroma and was not significantly different from controls. Determining the MND to the next tumor cell or keratinocyte confirmed the results of the cell density tests (**Figures 4B, C**). CD68^hi^CD163^lo^ and CD68^hi^CD163^hi^ cells of the CIS and HNSCC groups showed significantly lower MND compared to the control group, while no clear differences were measurable for CD68^lo^CD163^hi^ cells and T cells (**Figure 4B**). Distance differences between the different cell classes were detectable in the HNSCC group, but not in the CIS or control group. Within HNSCC, MND to the nearest tumor cell was significantly lower in the CD68^hi^CD163^lo^ subset compared with the CD68^hi^CD163^hi^ and CD68^lo^CD163^hi^ subsets as well as with T cells (**Figure 4C**).

**Figure 5:**
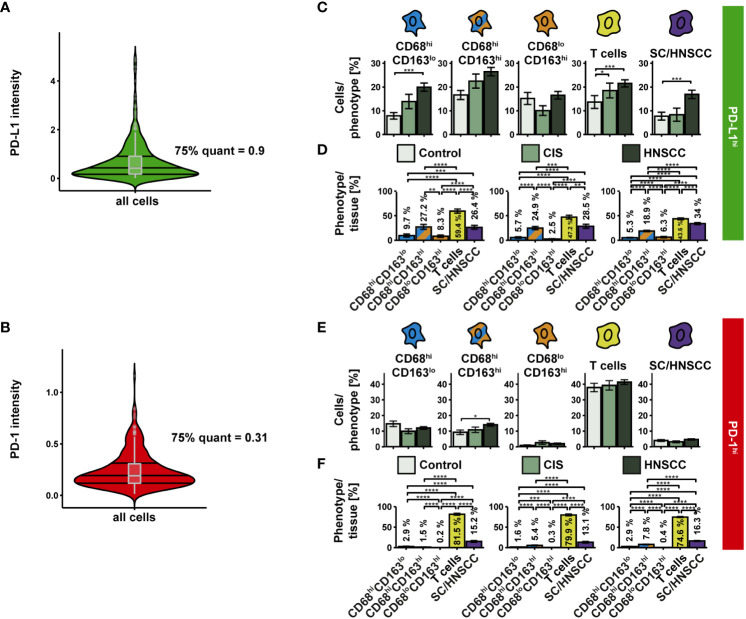
PD-L1 and PD-1 expression on CD68/CD163 subsets, T cells, and SC/HNSCC in TMAs of controls, CIS and HNSCC. **(A, B)** PD-L1 and PD-1 were determined based on their thresholds. **(C)** Proportion of PD-L1^hi^ cells in relation to each cell type. **(D)** PD-L1^hi^ cells relative to all PD-L1^hi^ cells. **(E)** Proportion of PD-1^hi^ cells in relation to each cell type. **(F)** PD-1^hi^ cells relative to all PD-1^hi^ cells. Each bar reflects the averaged cell type expression rates (CD68^hi^CD163^lo^ cells, CD68^hi^CD163^hi^ cells, CD68^lo^CD163^hi^ cells, T cells and SC/HNSCC) color-coded by tissue type or cell type. The error bars represent the standard error. The significance (by Wilcoxon rank sum test) levels between the groups are indicated as asterisks above the plots. *p<0.05, **p<0.01, ***p<0.001, ****p<0.0001.

We then examined the expression of PD-L1 and PD-1 on the three CD68/CD163 subsets, T cells, and SC/HNSCC (**Figure 5**). PD-L1^hi^ and PD-1^hi^ cells were determined based on the mean intensity/cell (**Figures 5A, B**). We observed an increased relative PD-L1 content within CD68^hi^CD163^lo^ cells, T cells, and tumor cells or keratinocytes in HNSCC compared with controls (**Figure 5C**). Significant differences between HNSCC and CIS were not observed. Considering the total number of PD-L1-expressing cells in the tissues, T cells had the highest proportion of PD-L1-expressing cells, followed by tumor cells or keratinocytes and CD68^hi^CD163^hi^ cells (**Figure 5D**). In contrast to PD-L1, relative PD-1 content was increased in HNSCC compared with controls exclusively in CD68^hi^CD163^lo^ cells. (**Figure 5E**). Significant differences between HNSCC and CIS were again not observed. Overall, T cells had the highest proportion of PD-1-expressing cells to total PD-1-expressing cells, followed by tumor cells or keratinocytes and CD68^hi^CD163^hi^ cells (**Figure 5F**).

**Figure 6:**
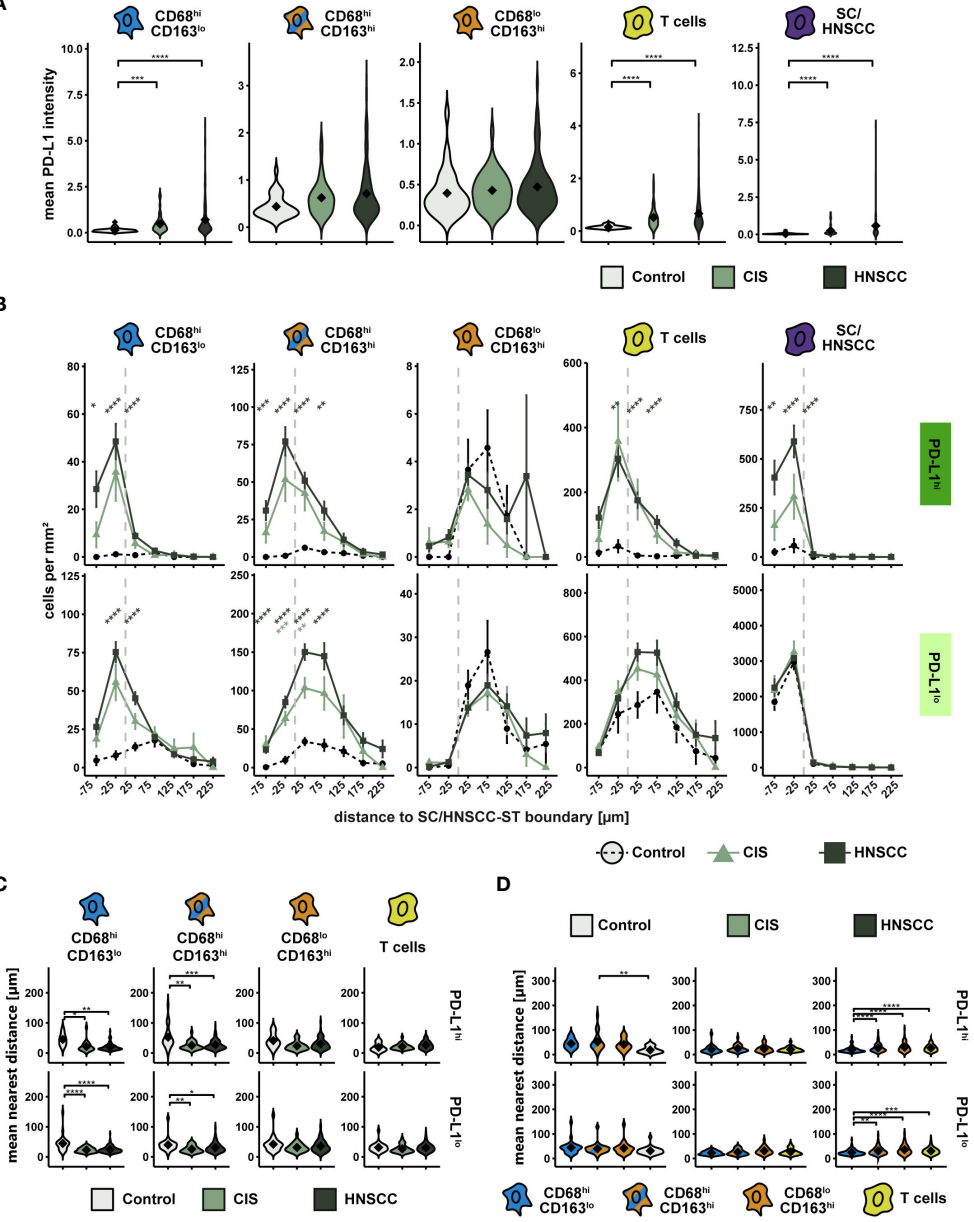
PD-L1 intensity and spatial relationships between PD-L1^hi^ cells in TMAs of controls, CIS and HNSCC. **(A)** PD-L1-intensity per cell type. Each violin plot reflects the averaged cell type PD-L1 (CD68^hi^CD163^lo^, CD68^hi^CD163^hi^ and CD68^lo^CD163^hi^, T cells and SC/HNSCC) subdivided and color-coded by tissue type (control, CIS and HNSCC). The significance (by Wilcoxon rank sum test) levels between the groups are indicated as asterisks above the plots. *p<0.05, **p<0.01, ***p<0.001, ****p<0.0001. **(B)** Cell density of PD-L1^hi/lo^ cells as a function of distance to SC/HNSCC-ST boundary. The distance to the SC/HNSCC-ST boundary or to the squamous epithelium is plotted on the X-axis. The vertical dashed line indicates the border between the stroma and the tumor or squamous epithelium. Negative X values reflect distances to SC/HNSCC-ST boundary within the tumors or squamous epithelium, positive X values distances in the adjacent stroma. Each line represents a group (control = dashed black, CIS = light green or HNSCC = dark green), each point represents the mean value of multiple samples. The error bars represent the standard error. The significance levels (by Wilcoxon rank sum test) between the groups are color-coded and indicated vertically as asterisks above the mean values. *p<0.05, **p<0.01, ***p<0.001, ****p<0.0001. **(C, D)** Nearest distance of PD-L1^hi/lo^ cell types to tumor cell or keratinocyte. Each violin plot reflects the averaged nearest distances of CD68/CD163 subsets (CD68^hi^CD163^lo^, CD68^hi^CD163^hi^ and CD68^lo^CD163^hi^) and T cells (CD3^+^) to next tumor cell or keratinocyte of the individual samples subdivided by tissue type (control, CIS and HNSCC). For better illustration, these have been sub sorted and color-coded by tissue type **(C)** or cell type **(D)**. The Y-axis reflects the distance to the nearest tumor cell or keratinocyte. The mean values are shown as diamonds. The significance (by Wilcoxon rank sum test) levels between the groups are indicated as asterisks above the plots. *p<0.05, **p<0.01, ***p<0.001, ****p<0.0001.

The highest PD-L1 intensity as well as the highest range between groups was observed within CD68^hi^CD163^lo^ MΦ and HNSCC (**Figure 6A**). Likewise, PD-L1 was upregulated on T cells. In contrast, no significant expression differences were detectable in the other two CD68/CD163 subsets. We then considered the cell density of PD-L1^hi^ and PD-L1^lo^ cells as a function of distance to SC/HNSCC-ST boundary (**Figure 6B**). In HNSCC and CIS, a significantly increased cell density of PD-L1^hi^ CD68^hi^CD163^lo^ cells, CD68^hi^CD163^hi^ cells and T cells was found in the tumor cell nests and in the immediate vicinity of the tumor cell border compared to controls. In these subsets the PD-L1^hi^ fraction was more concentrated in tumor cell nests compared to the PD-L1^lo^ fraction. PD-L1^hi^ CD68^lo^CD163^hi^ cells were found almost exclusively in the stroma. No differences between HNSCC, CIS and controls were detected here. In line with the cell density studies, we observed that the MND between PD-L1^hi^ CD68^hi^CD163^lo^ and CD68^hi^CD163^hi^ cells to the nearest tumor cell or keratinocyte was significantly lower in the CIS and HNSCC group compared to the control group (**Figure 6C**). In contrast, there were no differences in the MND of CD68^lo^CD163^hi^ cells and T cells. Significant MND differences between the CD68/CD163 subsets were observed mainly in the HNSCC group (**Figure 6D**). As already observed for the total population, the PD-L1^hi^ fraction of CD68^hi^CD163^lo^ subset also showed significantly lower MND to next tumor cell compared to the CD68^hi^CD163^hi^ and CD68^lo^CD163^hi^ subset. Thus, PD-L1 is expressed by multiple cell types in HNSCC. These include HNSCC, T cells and the CD68/CD163 subsets. Within the CD68/CD163 subsets, in particular CD68^hi^CD163^lo^ and CD68^hi^CD163^hi^ cells express PD-L1. Spatially, PD-L1 expression is increased especially in the SC/HNSCC-ST boundary region.

### Spatial relationship between PD-1-expressing T cells and PD-L1-expressing cells

**Figure 7:**
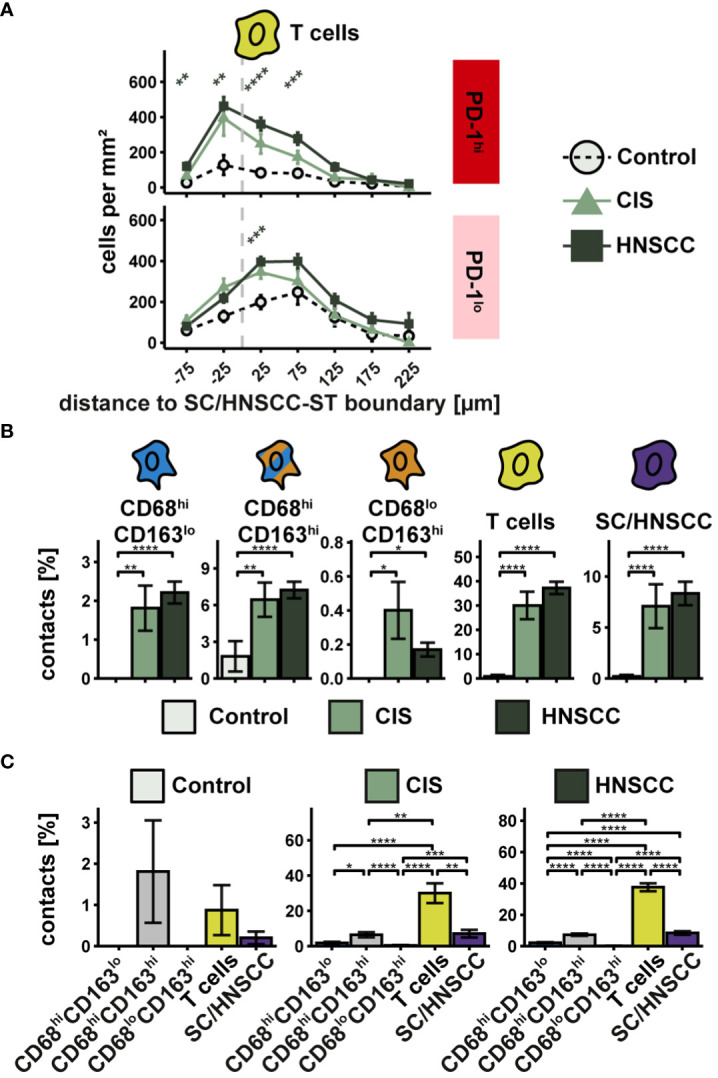
Spatial relationships between PD-1-expressing T cells and PD-L1-expressing cells in TMAs of controls, CIS and HNSCC. **(A)** Cell density of PD-1^hi^ T cells as a function of distance to SC/HNSCC-ST boundary. The distance to the SC/HNSCC-ST boundary or to the squamous epithelium is plotted on the X-axis. The vertical dashed line indicates the border between the stroma and the tumor or squamous epithelium. Negative X values reflect distances to SC/HNSCC-ST boundary within the tumors or squamous epithelium, positive X values distances in the adjacent stroma. Each line represents a group (control = black, CIS = light grey or HNSCC = dark grey), each point represents the mean value of multiple samples. The error bars represent the standard error. The significance levels (by Wilcoxon rank sum test) between the groups are color-coded and indicated vertically as asterisks above the mean values. *p<0.05, **p<0.01, ***p<0.001, ****p<0.0001. **(B, C)** Cell contacts of PD-1hi T cells with PD-L1hi cells. B Cell contacts PD-1^hi^ T cells with PD-L1^hi^ cells. Each bar indicates the averaged cell contacts of a cell type (CD68^hi^CD163^lo^ cells, CD68^hi^CD163^hi^ cells and CD68^lo^CD163^hi^ cells, T cells, and SC/HNSCC) as a percentage per T cell, sub sorted by cell type **(B)** and tissue **(C)** for improved overview. Color-coding was based on cell type. The error bars represent the standard error. The significance (by Wilcoxon rank sum test) levels between the groups are indicated as asterisks above the plots. *p<0.05, **p<0.01, ***p<0.001, ****p<0.0001.

PD-L1 exerts its inhibitory effect by interacting with PD-1 on T cells. The extent of interactions of PD-L1^hi^ cells with PD-1^hi^ T cells thus provides an indication of the extent of inhibitory activity via PD-L1/PD-1. To investigate the spatial relationship between PD-L1 and PD-1 expressing cells, we first examined the cell density of PD-1^hi^ T cells as a function of distance to SC/HNSCC-ST boundary (**Figure 7A**). Since PD-L1 expressing CD68/CD163 subsets localized on average very close to the SC/HNSCC-ST boundary, an increased density of PD-1 expressing T cells in the vicinity of the SC/HNSCC-ST boundary would favor PD-L1/PD-1 interaction. Indeed, the density of PD-1^hi^ T cells in CIS or HNSCC was significantly elevated in the tumor nests and close to the border compared to control. Compared to the PD-1^lo^ T cells the density peak was also more condensed in the tumor nests. Next, we determined what proportion of PD-1-expressing T cells had contact with PD-L1-expressing CD68/CD163 subsets and tumor cells (**Figures 7B, C**). We found significantly more cell-cell contacts to PD-1-expressing T cells in CIS and HNSCC compared with controls in all 5 pre-described cell subsets (**Figure 7B**). The differences between controls and CIS or HNSCC were most pronounced in the CD68^hi^CD163^lo^ cells. In absolute terms, T cells followed by tumor cells and CD68^hi^CD163^hi^ cells represented the highest proportion of cell contacts with T cells (**Figure 7C**). CD68^lo^CD163^hi^ cells accounted for the least amount of cell contact with T cells. PD-1 is thus expressed on T cells mainly at the SC/HNSCC-ST boundary, and these cells are in spatial proximity to PD-L1^hi^ cells. Furthermore, we demonstrated that the number of cell-cell contacts between PD-1^hi^ T cells and PD-L1^hi^ cells was significantly increased in CIS and HNSCC compared to control tissues, suggesting localized immunosuppression through the PD-L1/PD-1 pathway in the context of tumorigenesis. Within the CD68/CD163 subsets, this mainly affected CD68^hi^CD163^lo^ and CD68^hi^CD163^hi^ cells, further supporting our hypothesis of a different function of the subsets defined by CD68 and CD163.

### Association between spatial cell localization and clinical outcome

**Figure 8:**
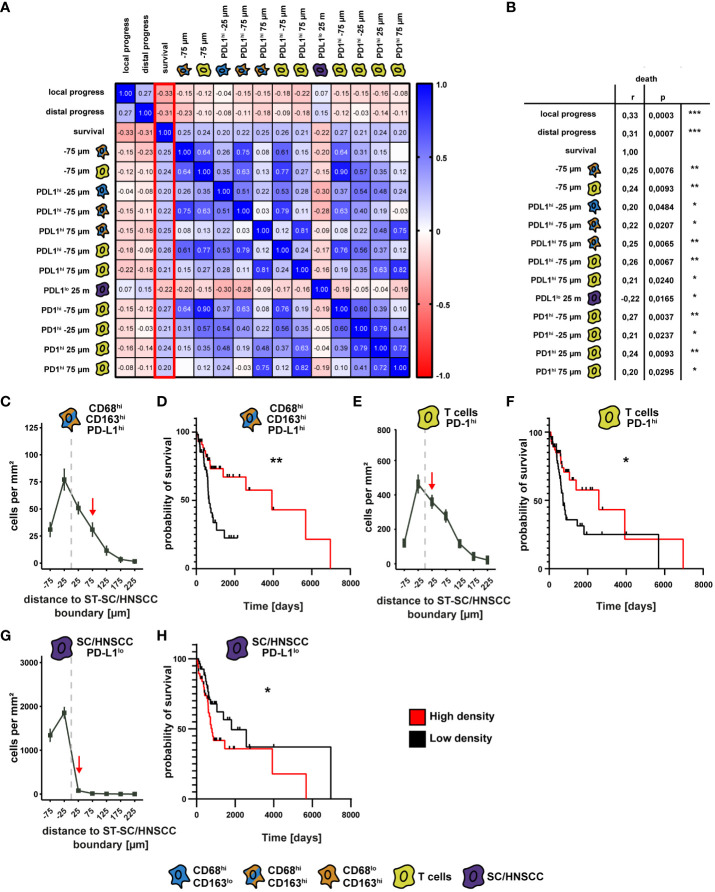
Comparison of spatial cell-cell relationships with clinical outcome. **(A)** Correlation matrix as a heat map of survival with cell density as a function of distance to squamous epithelium (SC) or HNSCC-stroma (ST) boundary (SC/HNSCC-ST boundary) and the events “local tumor progression” and “distal tumor progression”. Here, the events that showed a significant correlation with survival (highlighted red) were selected. **(B)** Table of significant events correlating with survival. The significance levels (by Spearman’s correlation) between the groups are indicated as asterisks above the plots. *p<0.05, **p<0.01, ***p<0.001, ****p<0.0001. **(C)** Cell density of CD68^hi^CD163^hi^PD-L1^hi^ cells as a function of distance to ST-CC/HNSCC boundary. Each point represents the mean value of multiple samples. Negative X values reflect distances to SC/HNSCC-ST boundary within the tumors or squamous epithelium, positive X values distances in the adjacent stroma. The vertical dashed line indicates the border between the stroma and the tumor or squamous epithelium. **(D)** Kaplan-Meier curves of patient survival with a high (> median) compared to a low (≤ median) density of CD68^hi^CD163^hi^PD-L1^hi^ cells at 75 µm from the ST-CC/HNSCC boundary. The significance levels (by Log-rank (Mantel-Cox) test) between the groups are indicated as asterisks above the plots. *p<0.05, **p<0.01, ***p<0.001, ****p<0.0001. **(E)** Cell density of PD-1^hi^ T cells as a function of distance to ST-CC/HNSCC boundary. Labeling and symbols as in C. **(F)** Kaplan-Meier curve of survival of patients with a high compared with a low density of PD-1^hi^ T cells at 25 µm from the ST-CC/HNSCC boundary. Labeling and symbols as in D. **(F)** Cell density of PD-L1^lo^ tumor cells as a function of distance to ST-CC/HNSCC boundary. Labeling and symbols as in **(D)**. **(G)** Cell density of PD-L1^lo^ tumor cells as a function of distance to ST-CC/HNSCC boundary. **(H)** Kaplan-Meier curve of survival rate of patients with a high compared to a low density of PDL1^lo^ tumor cells at 25 μm from the ST-CC/HNSCC boundary. Labeling and symbols as in **(D)**.

We subsequently evaluated if there is a correlation between spatial distribution of CD68/CD163 subsets, T cells and tumor cells to clinical outcome parameters (**Figure 8** and **Supplementary Figure 3**). In particular, a series of correlations emerged for cell density as a function of distance from the SC/HNSCC-ST boundary to events such as survival, local metastasis, and distal metastasis (**Supplementary Figure 3**). We focused on all events correlated with survival (**Figures 8A, B**). Here, we divided patients into high and low cell density cohorts based on the median cell density in each case and performed comparative survival time analyses. Thereby we identified three events that significantly affected survival. The most pronounced effect on survival occurred for CD68^hi^CD163^hi^PD-L1^hi^ cells at 75 µm from the SC/HNSCC-ST boundary. Here, the high cell density group showed significantly improved survival compared with the low cell density group (**Figures 8C, D**). When patients were divided into two groups based on threshold 0 (no PD-L1 expression versus any other expression level), the significance level was even higher (see **Supplementary Figures 3B, C**). In PD-1-expressing T cells at 25 µm from the SC/HNSCC-ST boundary, the high cell density group also showed improved survival compared with the low cell density group (**Figures 8E, F**). This was reversed within the PD-1^lo^ tumor cells at 25 µm from the SC/HNSCC-ST boundary. Here, the high cell density group showed poor survival compared to the low cell density group (**Figures 8G, H**). Overall, these data may argue for improved survival prognosis depending on PD-L1/PD-1 expression by specific cell types in proximity to SC/HNSCC-ST boundary. The poor survival at a higher cell density of PD-L1^lo^ tumor cells at 25 µm from the SC/HNSCC-ST boundary might be associated with increased invasiveness of tumor cells. Further correlations between spatial distribution and clinical parameters can be taken from **Supplementary Figure 3** and **Supplementary Table 8**.

### Characterization of the CD68/CD163 subsets in HNSCC via single cell RNA datasets

**Figure 9:**
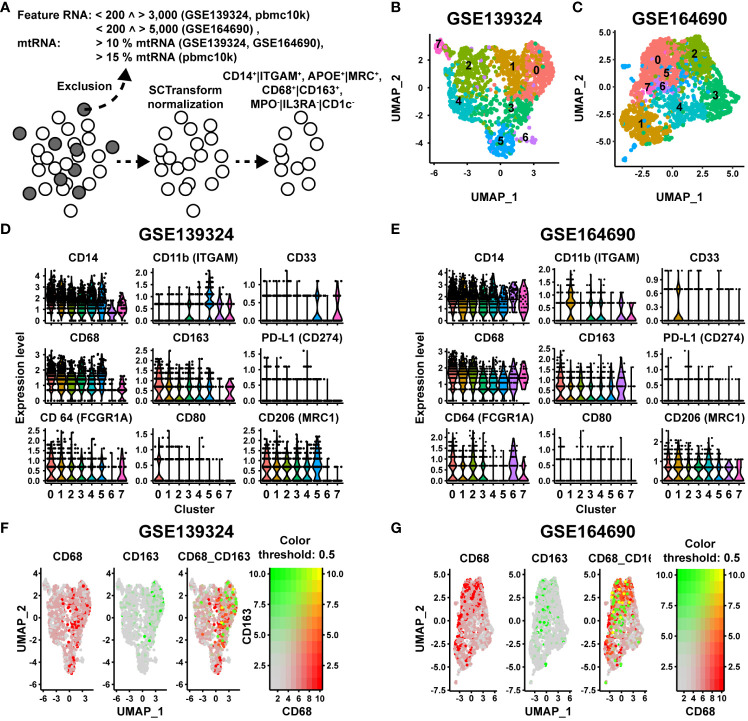
Single cell NGS analysis of CD68/CD163 MΦ clusters in 2 datasets (GSE139324 and GSE164690) of human HNSCC. **(A)** Quality control (QC), metrics and selection of CD14+|CD11b+, MPO-|CD123-|CD1C-, APOE+|MRC1+ cells before further analysis. **(B, C)** UMAP visualization of the CD68/CD163 MΦ clusters of human HNSCC (study A (GSE139324): 3446 cells, study B (GSE164690): 3930 cells). **(D, E)** Violin plots of the expression levels of CD14, CD11c, CD33, CD68, CD163, PD-L1, CD64, CD80 and CD206 within the clusters of A (GSE139324) and B (GSE164690). **(F, G)** Color-coded feature plot of CD68 and CD163 expression per cell projected onto the UMAP visualization of A (GSE139324) and B (GSE164690).

Besides MΦ, in particular monocytes and dendritic cells (DCs) express CD68 and CD163 (30, 41). CD68 is further expressed to varying degrees on granulocytes (42, 43). These cells cannot be distinguished from MΦ based on the IHC panel we used. To validate and further characterize the CD68/CD163 subsets as MΦ, we first examined single-cell mRNA datasets of two previously published studies in HNSCC patients for co-expression of CD68 and CD163 and other MΦ-specific markers. In study 1 (data set GSE139324 (9)), tumor-infiltrating leukocytes (TIL) from primary tumor tissue (n = 18) and CD45^+^ blood cells from immunotherapy-naïve HNSCC patients (18 HPV^-^, 8 HPV^+^), tonsil tissue from sleep apnea patients (n = 5), and CD45^+^ blood cells from healthy donors (n=6) were isolated. In study 2 (data set GSE164690 (17)), primary tumor tissue (12 HPV^-^, 6 HPV^+^) and PBL (n = 15) from immunotherapy-naïve HNSCC patients were isolated. CD45^+^ and CD45^-^ cells were FACS-sorted from the tumor tissue. We restricted ourselves to the presentation of the TIL of both single-cell RNA datasets. However, for quality assurance, we additionally compared the blood cell datasets with the 10X genomics dataset of PBMCs (Published on September 14th, 2021. This dataset is licensed under the Creative Commons Attribution license) (**Supplementary Table 9**). Before any filtering of cell types was performed, the datasets were first cleared of low-quality cells, empty droplets, cell duplicates, and cells with high levels of mitochondrial DNA, and were then normalized to remove this technical variability (**Figure 9A**). Monocytes and MΦ were then selected based on CD14 and/or CD11b. Additionally, DCs, monocytes and granulocytes were excluded via myeloperoxidase (MPO), CD123 and CD1c. MΦ were finally selected as CD68^+^ and/or CD163^+^. In analyzing the two HNSCC-single cell RNA datasets, we focused on CD68, CD163, and PD-L1. For further characterization, we also examined the expression of CD11b, CD14, CD33, CD64, CD80 and CD206. Since two raw datasets were processed using different CellRanger versions and gene annotation references, we restricted our analysis exclusively to the comparison of the two studies using published gene barcode matrices and did not perform any aggregation of the data. In both TIL datasets, UMAP analysis generated 8 distinct MΦ clusters (**Figures 9B, C**). The 10 most frequent cluster-defining genes of the respective clusters of both datasets are listed in **Supplementary Table 10**. Differences in the order or number of these genes defined for each cluster indicate a possible functional variability of these MΦ. CD14 and CD11b (ITGAM) were expressed in all MΦ clusters based on the previous filtering (**Figures 9D, E**). Compared with each other, CD14 was expressed by a higher proportion of cells in comparison with CD11b. CD33 was also expressed in all TIL clusters but as expected, in most clusters only by a fewer cell proportion, as its expression level decreases with cell maturation. In both TIL datasets, all MΦ clusters expressed CD68 and CD163. CD68 was generally expressed at a higher cell rate than CD163. Clusters with high CD68 rate and low CD163 rate or high CD68 and CD163 rate were found in both TIL datasets. Clusters with a higher CD163 expression rate compared to CD68 were only observed in blood samples from tumor patients (**Supplementary Figure 4**), but not in TIL. At the single-cell RNA expression level, we found CD68^hi^CD163^lo^, CD68^hi^163^hi^, CD68^lo^CD163^hi^ and CD68^lo^CD163^lo^ MΦ, with CD68^hi^CD163^hi^ showing the lowest percentage (**Figures 9F, G**). PD-L1 at the mRNA level was present in only a small proportion of cells. Therefore, correlation within specific clusters could not be determined. CD64 mRNA expression was found in all MΦ clusters. Compared to CD68, the proportion of CD64-expressing cells varied between clusters. CD80 was expressed by only a small proportion of MΦ. In contrast CD206 was expressed by a larger proportion of MΦ in most clusters. Thus, the CD68/CD163 subsets defined by IHC are reflected at the single-cell mRNA level within MΦ clusters. Overall, the single-cell mRNA data indicate a high functional specialization of these MΦ. However, the subtypes defined by CD68 and CD163 cannot be assigned to specific clusters. Due to the low number of PD-L1-positive cells a statistically significant correlation of PD-L1 to specific clusters was not possible. Notably, a predominant proportion of the MΦ expresses CD206 but not CD80 on mRNA level.

### Flow cytometric characterization of CD68/CD163 subsets in HNSCC

**Figure 10:**
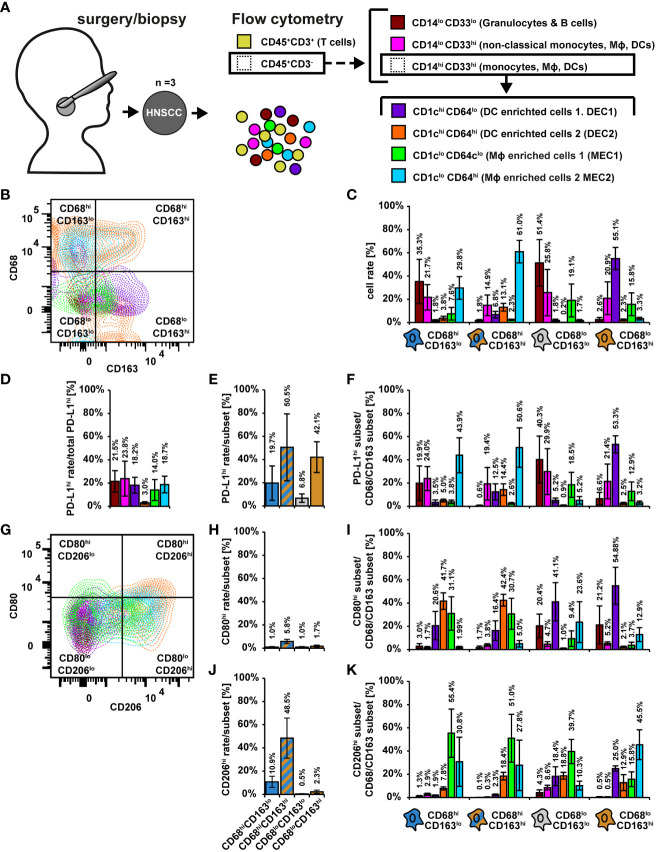
Flow cytometric characterization of CD68/CD163 subsets in HNSCC. **(A)** Schematic overview. Single cell suspensions of 3 primary human HNSCCs were labeled using fluorochrome-conjugated antibodies. Cell differentiation was performed according as shown. The detailed gating can be found in **Supplementary Figure 5**. **(B)** Exemplary dot plot of the distribution of leukocyte populations within CD68/CD163 subsets. **(C)** Proportion of leukocyte populations within the CD68/CD163 subsets. B Exemplary dot plot of the distribution of leukocyte populations within CD68/CD163 subsets. C Proportion of leukocyte populations within the CD68/CD163 clusters. **(D)** Proportion of PD-L1-expressing leukocytes to total PD-L1 expression. **(E)** Proportion of PD-L1 expressing cells within the CD68/CD163 subsets. **(F)** Proportion of PD-L1-expressing leukocyte subtypes to PD-L1 expression of the respective CD68/CD163 subset. **(G)** Exemplary dot plot of CD80 and CD206 expression of leukocyte populations. **(H)** Proportion of CD80 expressing cells within the CD68/CD163 subsets. **(I)** Proportion of CD80-expressing leukocytes to total CD80 expression within the CD68/CD163 subsets. **(J)** Proportion of CD206 expressing cells within the CD68/CD163 subsets. **(K)** Proportion of CD206-expressing leukocyte subtypes to CD206 expression of the respective CD68/CD163 subset. Within bar plots each bar represents the mean value of three samples. The error bars show the standard error.

The above discussed data support the view that ascribing of cell phenotypes based solely on mRNA expression data can be challenging (37, 44). We therefore used the mRNA datasets as a basis for further flow cytometric characterization of the CD68/CD163 subsets. We analyzed 3 primary HNSCCs by flow cytometry (**Figure 10**). After leukocyte pre-selection, doublet elimination, and T cell exclusion by CD3, CD45^+^CD3^-^ leukocytes were further divided by CD14 and CD33 into CD14^lo^CD33^lo^ (granulocytes and B cells) CD14^lo^CD33^hi^ (non-classical monocytes, MΦ, DCs) and CD14^hi^CD33^hi^ (monocytes, MΦ and DCs) (**Figure 10A** and **Supplementary Figures 5A–E**). CD14/CD33 subsets were further differentiated by CD1c (DCs, some monocytes and B cells) and CD64 (monocytes, MΦ neutrophil some granulocytes and DCs) as well as by HLA-DR (MΦ, DCs, some monocytes and B cells) and MerTK (mainly mature MΦ) (**Figure 10A** and **Supplementary Figures 5F–M**). A detailed explanation of the markers used for gating is provided in **Supplementary Figure 5**. As expected, most of the CD14^lo^CD33^lo^ cells expressed little to no CD1c and CD64, supporting that monocytes, MΦ or DCs were hardly present (**Supplementary Figure 5F**). By contrast the CD14^lo^CD33^hi^ cells could be divided into three populations, CD1c^hi^CD64^lo^, CD1c^lo^CD64^lo^ and CD1c^lo^CD64^hi^ (**Supplementary Figure 5G**) and the CD14^lo^CD33^hi^ into four populations, CD1c^hi^CD64^lo^, CD1c^hi^CD64^hi^, CD1c^lo^CD64^lo^ and CD1c^lo^CD64^hi^ (**Figure 10A**, **Supplementary Figure 5H**). Approximately 67% of the CD14^lo^CD33^lo^ cells expressed high and intermedium levels of HLA-DR (**Supplementary Figures 5I, J**) which is consistent with both B cells and granulocytes. About 28% expressed little to no HLA-DR. These cells are presumably granulocytes. MerTK was expressed by a negligible number of CD14^lo^CD33^lo^ cells. The majority of the CD14^lo^CD33^hi^ and CD14^hi^CD33^hi^ were HLA-DR^hi^, and some were also MerTK^hi^ (**Supplementary Figures 5K–N**). This suggests that they predominantly consist of mature MΦ or DCs. To simplify matters, we named the CD1c^hi^ subsets DC-enriched cells (DEC1, CD1c^hi^CD64^lo^, DEC2, CD1c^hi^CD64^hi^) and the CD1c^lo^ subsets MΦ-enriched cells (MEC1, CD1c^lo^CD64^lo^, MEC2, CD1c^lo^CD64^hi^). In our further considerations we distinguished the following 6 populations CD14^lo^CD33^lo^ (granulocytes and B cells), CD14^lo^CD33^hi^ (non-classical monocytes, MΦ, DCs), (CD14^hi^CD33^hi^)CD1c^hi^CD64^lo^ (DC-enriched cells 1, DEC1), (CD14^hi^CD33^hi^)CD1c^hi^ CD64^hi^ (DC-enriched cells 2, DEC2), (CD14^hi^CD33^hi^)CD1c^lo^CD64^lo^ (MΦ-enriched cells 1, MEC1) and (CD14^hi^CD33^hi^)CD1c^lo^CD64^hi^ (MΦ-enriched cells 2, MEC2) (**Figure 10A**).

We determined the proportion of the mentioned leukocyte populations within the CD68/CD163 clusters (**Figures 10B, C** and **Supplementary Figures 5O–Q**). The CD68^hi^CD163^lo^ subset was mainly composed of CD14^lo^CD33^lo^ cells (35.3%), CD14^lo^CD33^hi^ cells (21.7%), MEC1 (29.8%) and MEC2 (7.6%). The CD68^hi^CD163^hi^ subset was mainly formed by MEC1 (61.0%), followed by CD14^lo^CD33^hi^ cells (14.9%) and DEC2 (13.1%). In the CD68^lo^CD163^hi^ subset, we detected mainly DEC1 (55.1%), followed by CD14^lo^CD33^hi^ cells (20.9%) and MEC2 (15.8%), and in the CD68^lo^CD163^lo^ subset CD14^lo^CD33^lo^ cells (51.4%), followed by CD14^lo^CD33^hi^ cells (25.8%) and DEC2 (19.1%). Overall, these results suggest that the CD68^hi^CD163^lo^ subset defined by IHC consists predominantly of MΦ and monocytes (MEC1, MEC2, CD14^lo^CD33^hi^ cells), but may also contain a significant proportion of granulocytes. In contrast, the CD68^hi^CD163^hi^ subset is predominantly composed of mature MΦ, followed by monocytes and DCs. The CD68^lo^CD163^hi^ subset appears to be formed to a significant extent by DCs, but also by monocytes and MΦ.

In the following, we examined PD-L1 expression within the leukocyte subsets (**Figures 10D–F**). In DEC2, only a small proportion expressed PD-L1 (**Figure 10D**). The remaining cells contributed to PD-L1 expression in approximately equal proportions (14 – 24%). We next determined the proportion of PD-L1 expressing cells within the CD68/CD163 subsets (**Figure 10E**). Within the CD68/CD163 subsets, we observed the highest proportion of PD-L1-expressing cells in the CD68^hi^CD163^hi^ (50.5%) subset, followed by the CD68^lo^CD163^hi^ subset (42.1%). We then determined the proportion of PD-L1-expressing leukocyte subtypes to PD-L1 expression of the respective CD68/CD163 subset (**Figure 10F**). Within the CD68^hi^CD163^lo^ subset, the highest proportion of PD-L1-expressing cells were MEC2 (43.9%), followed by CD14^lo^CD33^hi^ cells (24.0%) and CD14^lo^CD33^lo^ cells (19.9%). MEC2 also were the major PD-L1 expressing cells (50.6%) within the CD68^hi^CD163^hi^ subset, followed by CD14^lo^CD33^hi^ cells (19.4%) DEC2 (14.4%) and DEC1 (12.5%). Within the CD68^lo^CD163^hi^ subset, DEC1 cells (53.3%) were the major PD-L1-expressing cells, followed by CD14^lo^CD33^hi^ cells (21.4%) and MEC2 (12.9%). The main PD-L1 expressing cells within the CD68^lo^CD163^lo^ subset were CD14^lo^CD33^lo^ cells (40.3%), followed by CD14^lo^CD33^hi^ cells (29.9%) and MEC1 (18.5%). Thus, PD-L1 appears to be primarily expressed by monocytes/MΦ within the CD68^hi^CD163^lo^ and CD68^hi^CD163^hi^ subsets, whereas within the CD68^lo^CD163^hi^ subset PD-L1 is expressed to a considerable extent by DCs, but also monocytes/MΦ.

We further characterized the proportion of CD80- and/or CD206-expressing cells within the leukocyte populations (**Figures 10G–K**). On MΦ CD80 is associated with a proinflammatory (M1) phenotype (Jaguin et al., 2013; Tarique et al., 2015; Rey-Giraud et al., 2012; Valdez et al., 2022), whereas CD206 is expressed on average more on M2-MΦ (Jaguin et al., 2013; Valdez et al., 2022; Rey-Giraud et al., 2012; Larionova et al., 2020). Overall, only a small proportion of CD45^+^CD3^-^ leukocytes expressed CD80 (**Figure 10G**). Within the CD68^hi^CD163^lo^ and CD68^hi^CD163^hi^ subgroups (**Figure 10H**), the major CD80-expressing cells were DEC1 (47.3% and 42.4%), followed by MEC1 (31.1% and 30.7%) and MEC1 (20.6% and 16.4%). In addition, within the CD68^lo^CD163^lo^ and CD68^lo^CD163^hi^ subgroups, the major CD80-expressing cells were DEC1 (44.1% and 54.9%), followed by CD14^lo^CD33^lo^ cells (20.4% and 21.2%) and MEC2 (23.6% and 12.9%). CD206 was expressed by a relatively high proportion of CD45^+^CD3^-^ leukocytes (**Figure 10G**). The CD68^hi^CD163^hi^ subset (48.5%) had the highest proportion of CD206-expressing cells (**Figure 10J**), followed by the CD68^hi^CD163^lo^ subset (10.9%). Within the CD68^hi^CD163^lo^ and CD68^hi^CD163^hi^ subsets (**Figure 10K**), the major CD206-expressing cells were MEC1 (55.4% and 51.1%), followed by MEC2 (30.8% and 27.8%) and DEC2 (7.9% and 18.4%). Within the CD68^lo^CD163^lo^ subset, MEC1 (39.7%) also were the major CD206-expressing cells, followed by DEC2 (18.8%), DEC1 (18.4%), and MEC2 (10.3%). In contrast, the major CD206-expressing cells within the CD68^lo^CD163^hi^ subset were MEC2 (45.5%), followed by DEC1 (25.0%). These results suggest that in HNSCC, CD80 is predominantly expressed by DCs and CD206 is predominantly expressed by MΦ, which is supported by the single cell RNA data in which the MΦ clusters clearly predominantly express CD206 but hardly express CD80.

To better classify the expression profiles detected in HNSCC, we validated the flow cytometric assays on CD14-MACS-enriched monocyte-derived M0, M1, and M2 MΦ (MDMs) (**Supplementary Figure 6**). Via CD14/CD33 selection (**Supplementary Figures 6A–E**), we obtained exclusively CD14^hi^CD33^hi^ cells (**Supplementary Figure 6E**). Upon further differentiation using CD1c and C64, the M0 and M1 MDMs were found to be CD64^hi^, whereas the M2 MDMs were CD64^lo^ (**Supplementary Figure 6K**). CD1c was not expressed by any of the three MΦ differentiation types, which is consistent with the hypothesis that the CD1c^hi^ subsets detected in the tumor were DCs. M1 MDMs were almost entirely HLA-DR^hi^ and partially MerTK^hi^. whereas M0 and M2 MDMs exhibited a higher proportion of HLA-DR^lo^ cells (**Supplementary Figure 6L**). M0 MDMs showed the highest proportion of HLA-DR^hi^MerTK^hi^ cells. All three differentiation types were predominantly CD68^hi^CD163^hi^, with a minor proportion of CD68^lo^CD163^hi^ (**Supplementary Figure 6M**). M1 MDMs had a slightly higher proportion of CD68^lo^CD163^hi^ cells compared to M0 and M2 MDMs. PD-L1 was expressed by both, M1 and M2 MDMs, but not by M0 MDMs (**Supplementary Figure 6N**). A small proportion of M0 and M1 MDMs expressed CD80 (**Supplementary Figure 6O**). As expected M2 MDMs did not express CD80. However, consistent with the literature, M2 MDMs had a higher CD206^hi^ content compared with M0 and M1 MDMs. We next investigated whether intracellular CD163 could lead to a discrepancy between IHC data and FC data (**Supplementary Figure 7**). A mixture of M0, M1, and M2 MDMs were therefore labeled extracellularly and intracellularly with differentially conjugated CD163 antibodies from the same clone, among others. This showed that intracellular CD163 was detected exclusively when CD163 was also expressed extracellularly. Thus, the CD68^hi^CD163^lo^ and CD68^hi^CD163^hi^ subsets in HNSCC defined by IHC predominantly consist of MΦ and monocytes. The CD68^lo^CD163^hi^ subset contains, to a considerable extent, DCs. With the exception of DEC2 and T cells, the proportion of PD-L1-expressing cells was approximately equal in all subsets determined by FC. PD-L1-expressing CD68^hi^CD163^lo^ and CD68^hi^CD163^hi^ cells were mainly MΦ. In contrast, PD-L1-expressing CD68^lo^CD163^hi^ cells were mainly DCs. CD64 is not expressed by all monocytes/MΦ in HNSCC. Our studies of MDMs suggest that some of these are M1-polarized MΦ, but based on CD68, CD80, CD163, and CD206, which are typically used for MΦ polarization, it is apparent that tumor-associated MΦ subsets occupy a broad spectrum within this classification.

## Discussion

Our immunohistochemical studies on HNSCC clearly indicated a heterogeneity of CD68 and CD163 distribution and cells corresponding thereto. This was confirmed by our spatial studies on cells that we distinguished based on CD68/CD163 expression levels. Here, we differentiated three subsets that were either CD68^hi^CD163^lo^, CD68^hi^CD163^hi^, and CD68^lo^CD163^hi^ cells. Cell density and nearest-distance determinations showed that the three subsets resided spatially predominantly in different areas of the tumor microenvironment. CD68^hi^CD163^lo^ cells and CD68^hi^CD163^hi^ cells were mainly found near the SC/HNSCC-ST boundary, whereas CD68^lo^CD163^hi^ cells were relatively uniformly distributed in the stroma and, on average, were significantly farther from tumor cells. Comparison to healthy tissue and CIS also strongly supports a tumor-specific origin of the observed distribution patterns. We therefore addressed the question of whether the CD68/CD163 subsets are functionally distinct from each other and whether the spatial relationships may provide additional insight. Kürten et al. (17) showed by multiplex IHC that CD68^+^ MΦ expressed PD-L1 at the SC/HNSCC-ST boundary and within the tumor nests. In addition, they detected T cells near the SC/HNSCC-ST boundary. However, they did not differentiate between CD68 and CD163. They also did not include PD-1 in their multiplex IHC analysis. Another study on HNSCC examined PD-1 expression on different T-cell subsets within tumor nests. They found that PD-1^+^ T-helper and T_REG_ cell density as well as colocalization of PD-1^+^ T-helper cells with CD163^+^ MΦ negatively correlated with overall survival (45). However, they did not further characterize CD163^+^ MΦ for other MΦ markers or PD-L1. We therefore simultaneously examined the spatial relationships of PD-1 and PD-L1 on T cells, CD68/CD163 MΦ subsets, and HNSCC using multiplex IHC. Overall, the largest proportion of PD-L1-expressing cells was composed of leukocytes and tumor cells. Within CD68/CD163 subsets, the CD68^hi^CD163^hi^ cells showed the highest degree of PD-L1 expression. However, beside tumor cells, we found the highest PD-L1 intensity and also the highest difference between tumor samples and controls in the CD68^hi^CD163^lo^ subset. These results suggest differential dynamics with respect to PD-L1 expression on the different CD68/CD163 subsets. Further evidence for this was provided by our spatial studies. These suggest that PD-L1/PD-1 interactions in HNSCC occur predominantly in close proximity to the SC/HNSCC-ST boundary, as PD-L1^hi^ and PD-1^hi^ cells predominantly accumulated in and around the SC/HNSCC-ST boundaries. This was predominantly related to CD68^hi^CD163^lo^ cells, CD68^hi^CD163^hi^ cells, and T cells with increased cell-cell contacts with PD-1-expressing T cells in CIS and HNSCC. In contrast, we observed very few cell-cell contacts between PD-1-expressing T cells and CD68^lo^CD163^hi^ cells, which may indicate less involvement of these cells in PD-L1/PD-1-mediated T cell inactivation compared to the previously described subsets. Our clinical data underscore the importance of localization of PD-L1/PD-1 signaling and by which cell types it is mediated. Here, PD-L1-expressing CD68^hi^CD163^hi^ joined PD-1-expressing T cells near the SC/HNSCC-ST boundary and one might infer this results in T cell inhibition in association with poor survival. But the opposite was the case. Here, it is clear that a more sophisticated understanding of PD-L1/PD-1 interactions is needed. It is important to emphasize in this context, that beside T cells, also the CD68/CD163 subsets and tumor cells expressed PD-1 to some extent. Within the CD68/CD163 subsets, PD-1 was predominantly expressed by the CD68^hi^CD163^hi^ and CD68^hi^CD163^lo^ subsets. This suggests signaling via the PD-L1/PD-1 pathway in multiple directions. Few literature exists regarding PD-1 expression by monocytes/MΦ. Studies in mouse models and humans indicate increasing MΦ PD-1 expression with tumor progression (46). In these studies, PD-1 expression on MΦ negatively correlated with phagocytic potency against tumor cells. PD-1-PD-L1 blockade, in turn, resulted in an increased phagocytosis rate *in vivo*. PD-L1 expression on T cells also appears to be tumor-promoting via multiple pathways including an alternative M2-like program in the PD-1^+^ MΦ (47). Intrinsic PD-1 expression by tumor cells was also described for several tumor entities (48–50). This can lead to tumor-inhibiting and tumor-promoting effects, positively and negatively influencing checkpoint immunotherapy.

In immunohistochemical studies, CD68- and CD163-positive cells are often equated with MΦ. However, our and many other studies have clearly shown that both markers are also expressed by other cell types. Validation of our CD68/CD163 IHC subsets on two HNSCC scRNA-seq-datasets of two previously published studies reflected CD68/CD163 subsets defined by IHC at the single-cell mRNA level after strict MΦ preselection. Moreover, these data indicated a large heterogeneity within tumor-associated MΦ (TAMs) in HNSCC. However, assignment of CD68/CD163 subsets as well as PD-L1 expression to the aforementioned clusters was not feasible, which was partly related to the relatively low initial MΦ count after appropriate prefiltering of the single cell data. Studies simultaneously examining mRNA and protein expression at the single cell level (37, 44) also clearly indicate that differentiation of cell phenotypes at the mRNA level alone can be imprecise. Among other things, one of these studies also demonstrated that PD-L1 was underrepresented in cells at the mRNA level (37). Our flow cytometric studies showed that CD68 and CD163 predominantly consisted of MΦ/monocytes and DCs. CD68^hi^CD163^lo^ and CD68^hi^CD163^hi^ cells predominantly were MΦ, the former may also contain granulocytes to some extent. CD68^lo^CD163^hi^ cells, on the other hand, appear to predominantly be DCs. Differentiation of CD68/CD163 subgroups based on CD64, CD80, CD163, and CD206, which are commonly used to study MΦ polarization (51–54), indicate a broad spectrum of TAM differentiation in HNSCC within the classical M1/M2 polarization. In this context, CD206 is expressed by a large fraction of MΦ, while CD80 is expressed hardly at all. Remarkably, CD80 is expressed by a large fraction of DCs, substantiating the hypothesis that the CD68/CD163-IHC subsets have distinct functions in the microenvironment of HNSCC. An integrative, high-dimensional single-cell protein and RNA study differentiated phenotypically and functionally distinct cDC2 subsets based on CD5, CD163, and CD14 expression, including a proinflammatory subset related to DC3s that increased in patients with systemic lupus erythematosus and correlate with disease activity (34). This might point to a more proinflammatory role of the DCs we observed in HNSCC.

Overall, our data demonstrate that the development of HNSCC is associated with changes in spatial cell-cell relationships. Spatial dynamics, single-cell RNA data, and flow cytometric analysis indicate heterogeneity and different functions of CD68/CD163 subsets. The CD68^hi^CD163^lo^ and CD68^hi^CD163^hi^ subsets mainly contain MΦ and are predominantly located in and around the tumor nests. They express PD-L1 and interact with PD-1-expressing T cells, among others. CD68^lo^CD163^hi^ cells predominantly consist of DCs, are relatively uniformly distributed in the tumor stroma, express relatively little PD-L1 compared with the other two CD68/CD163 subsets, and interact rarely with PD-1-expressing T cells. Notably, correlations with clinical parameters also suggest that interactions via the PD-L1/PD-1 pathway in HNSCC should be understood as a multidirectional interplay between different cell types, with the spatial location of those interactions appearing to play a critical role in tumor outcome. These findings may be useful for more precise prediction of tumor response to immune checkpoint blockade. The current standards for indication for pembrolizumab therapy are the Tumor Proportion Score (TPS) and Combined Positive Score (CPS). These are manually determined by the pathologist for exemplary tumor areas and then extrapolated to the entire tumor. Automated PD-L1 determination in the immediate vicinity of the tumor could provide a much more sensitive tool in this regard. Differentiation of PD-L1/PD-1-expressing cell types and cell contacts between PD-L1/PD-1-expressing cells could further increase sensitivity. Our study does not include patients receiving first-line therapy with immune checkpoint inhibitors. This cohort would need to be studied to validate whether the spatial localization of PD-L1 and PD-1 expressing cell types to each other influences response to immune checkpoint blockade. However, particularly in light of several ongoing neoadjuvant trials of immune checkpoint inhibitors in HNSCC, some of these questions may soon be answered.

## Data availability statement

The datasets presented in this study can be found in online repositories. The names of the repository/repositories and accession number(s) can be found below: https://www.ncbi.nlm.nih.gov/geo/, GSE139324 (9), GSE164690 (17).

## Ethics statement

Written informed consent was obtained from all patients and the study was approved by the ethics committees of the University Medical Center Göttingen (ethical vote 25/7/18), the institutional review board of the University Cancer Center (UCT) and the ethical committee at the University Hospital Frankfurt (project numbers: 4/09, SKH-1-2019). With regard to the single-cell mRNA datasets from Cillo et al. (GSE139324 (9)) and Kürten et al. (GSE164690 (17)) the investigators provided written informed consent for all human patient samples prior to donation. The studies were approved by the review board of the University of Pittsburgh Cancer Institute (protocol 99-069). 10x genomics dataset of PBMCs (Published on September 14th, 2021) is licensed under the Creative Commons Attribution license.

## Author contributions

CN and AW conceptualized and designed research, CN developed methodology. CN and VA performed experiments and acquired data, CN, AW, and IM analyzed data and interpreted results, AW, JG, SK, PS, and DB provided technical and material support. CN and AW supervised research and all authors participated in writing the manuscript. All authors contributed to the article and approved the submitted version.
