# Supplementary material for: Association between spatial distribution of leukocyte subsets and clinical presentation of head and neck squamous cell carcinoma
Source: Front Immunol. 2024 Jan 23;14:1240394. doi: 10.3389/fimmu.2023.1240394 (PMC10844964; doi:10.3389/fimmu.2023.1240394)
Supplement: Supplementary file 1 [file DataSheet_1.zip › Data Sheet 1.pdf]

Supplementary Material

Association between spatial distribution of leukocyte subsets and clinical presentation of head and neck squamous cell carcinoma

Christoph Netzer[1#], Vanessa von Arps-Aubert[1], Igor Mačinković[2], Jens von der Grün[3,4], Stefan Küffer[5], Philipp Ströbel[5], Andreas von Knethen[6], Andreas Weigert[2*] and Dirk Beutner[1*]

[1]Department of Otorhinolaryngology, Head and Neck Surgery, University Medical Center Göttingen, Göttingen, Germany
[2]Institute of Biochemistry I, Faculty of Medicine, Goethe-University Frankfurt, Frankfurt am Main, Germany,
[3]Department of Radiation Oncology, University Hospital Zurich and University of Zurich, Zurich, Switzerland,
[4]Department of Radiotherapy and Oncology, University Hospital Frankfurt, Frankfurt, Germany,
[5]Institute of Pathology, University Medical Center Göttingen, Göttingen, Germany,
[6]Department of Anesthesiology, Intensive Care Medicine and Pain Therapy, University Hospital Frankfurt, Goethe-University Frankfurt, Frankfurt am Main, Germany
Short title: Spatial leukocyte heterogeneity in HNSCC

*These authors contributed equally

#Correspondence:
Dr. Christoph Netzer, Department of Otorhinolaryngology, Head and Neck Surgery, University Medical Center Göttingen, Robert-Koch-Straße 40, D-37075 Göttingen, Tel: +49 551 3962200, Fax: +49 551 3963773; E-Mail: christoph.netzer@med.uni-goettingen.de.

SUPPLEMENTARY TABLES 1 – 3 Patient cohort composition and clinicopathologic parameters

See corresponding Excel file.

SUPPLEMENTARY TABLE 4 Antibody panel for multiplexed immune fluorescence staining on whole slides

Nr.	Antigen	Manufactor	Cat. Nr.	Host	Dilution	Opal	AR	Opal-Dil.
1	IL-1β	MABTECH	3416-3-250	Mouse	1/200	520	2	1/250
2	CD68	Dako	M0876	Mouse	1/100	100	2	1/200
3	NLRP3	Novus	NBP2-12446	Rabbit	1/250	690	1	1/250
4	CD163	Abcam	ab182422	Rabbit	1/250	250	2	1/200
5	CK	Mouse	Dako	3515	1/100	480	2	1/200
6	LYVE-1	R&D	AF2089	Goat	1/100	620	1	1/200
7	CD45	abcam	Ab10558	Rabbit	1/200	570	1	1/150
8	Ki-67	abcam	Ab16667	Rabbit	1/250	TSA-Dig	2	1/100
						780		1/75
	Spectral DAPI	AKOYA	FP1490					

SUPPLEMENTARY TABLE 5 Antibody panel for multiplexed immune fluorescence staining on TMA

Nr.	Antigen	Manufactor	Cat. Nr.	Host	Dilution	Opal	AR	Opal-Dil.
1	PD-1	abcam	ab137132	Rabbit	1/500	620	1	1/150
2	CD68	Dako	M0876	Mouse	1/100	690	2	1/150
3	PD-L1	Spring Bio.	M4422	Rabbit	1/100	520	1	1/100
4	CD3	Ventana	790-4341	Rabbit	1	570	1	1/150
5	CD163	Abcam	ab182422	Rabbit	1/250	480	2	1/150
6	CK	Dako	3515	Mouse	1/100	TSA-Dig	2	1/100
						780		1/75
	Spectral DAPI	AKOYA	FP1490					

SUPPLEMENTARY TABLE 6 Secondary antibodies

Antigen	Manufactor	Cat. Nr.	Dilution
Mouse	Dako	K4001	Rtu
Rabbit	Dako	K4003	Rtu
Goat	Dako	P0160	1/200

SUPPLEMENTARY TABLE 7 Staining protocol for Bond RX

	Step	Time	Temperature
	Dewax	Auto program	
	Wash	3 x 30 sec	RT
	H_2O_2	10 min	RT
	Wash	3 x 30 sec	RT
Antigen 1	AR	20 min	95° C
	Wash	3 x 30 sec	RT
	Prim. Antibody	30 min	RT
	Wash	3 x 30 sec	RT
	Sek. Antibody	10 min	RT
	Wash	3 x 30 sec	RT
	Opal	10 min	RT
	Wash	3 x 30 sec	RT
Antigen 2	AR	20 min	95° C
	Wash	3 x 30 sec	RT
	Prim. Antibody	30 min	RT
	Wash	3 x 30 sec	RT
	Sek. Antibody	10 min	RT
	Wash	3 x 30 sec	RT
	Opal	10 min	RT
	Wash	3 x 30 sec	RT
Antigen 3 – x			
Last Antigen	AR	20 min	95° C
	Wash	3 x 30 sec	RT
	Prim. Antibody	30 min	RT
	Wash	3 x 30 sec	RT
	Sek. Antibody	10 min	RT
	Wash	3 x 30 sec	RT
	TSA-DIG	10 min	RT
	Wash	3 x 30 sec	RT
	Opal 780	10 min	RT
	Opal 780	60 min	RT
	Wash	3 x 30 sec	RT

Links to the phenoptr functions:

https://akoyabio.github.io/phenoptr/
https://akoyabio.github.io/phenoptr/reference/density_at_distance.html
https://akoyabio.github.io/phenoptr/articles/computing_distances.html
https://akoyabio.github.io/phenoptr/articles/find_and_count_touching_cells.html

Thresholds for PD-L1 and PD-1 expression

The threshold here was set as 75% quantile (75% quantile/slide (mean (intensity/cell))). Cells above the threshold were designated PD-L1^{hi} and PD-1^{hi}, respectively, and cells below the threshold were designated PD-L1^{lo} and PD-1^{lo}, respectively.

SUPPLEMENTARY TABLE 8 Data to Figure 3A: Correlation of clinical events with cell density as a function of distance to squamous epithelium (SC) or HNSCC-stroma (ST) boundary (SC/HNSCC-ST boundary)

See corresponding Excel file.

SUPPLEMENTARY TABLE 9 Comparison of cell counts before and after quality control (QC), metrics and selection of $CD14^{+}|CD11b^{+}$, $MPO^{-}|CD123^{-}|CD1C^{-}$, $APOE^{+}|MRC1^{+}$. Comparison of HNSCC single-cell RNA datasets (1, 2)and 10X genomics dataset on PBMCs (Published on September 14th, 2021. This dataset is licensed under the Creative Commons Attribution license). Each column describes corresponding data sets of the different studies.

A Cillo et al.

	HD_PBMC		HD_Tonsil		HNSCC_PBMC		HNSCC_TIL	
	# of cells	% of cells	# of cells	% of cells	# of cells	% of cells	# of cells	% of cells
**Detected cells**	12.244	100,0	12.898	100,0	47.106	100,0	60.932	100,0
**Cells after QC**	11.971	97,8	12.502	96,9	45.476	96,5	57.511	94,4
**CD14+ | CD11b+**	2.559	20,9	153	1,2	13.517	28,7	7.197	11,8
**MPO- | CD123- | CD1C-**	2.398	19,6	130	1,0	12.677	26,9	6.086	10,0
**APOE+|MRC1+**							1.699	2,8
**CD68+ or CD163+**	1.161	9,5	33	0,3	6.736	14,3	1.372	2,3
**T cells (CD8A)**	1.395	11,4	578	4,5	5.599	11,9	14.248	23,4
**T cells (IL7R)**	4.295	35,1	3.294	25,5	12.382	26,3	15.323	25,1

B Kürten et al.

	PBL		CD45p	
	# of cells	% of cells	# of cells	% of cells
**Detected cells**	36.030	100,0	64.711	100,0
**Cells after QC**	34.372	95,4	60.272	93,1
**CD14+ | CD11b+**	9.736	27,0	7.331	11,3
**MPO- | CD123- | CD1C-**	9.156	25,4	6.209	9,6
**APOE+|MRC1+**			2.262	3,5
**CD68+ or CD163+**	4.839	13,4	1.985	3,1
**T cells (CD8A)**	2.757	7,7	13.399	20,7
**T cells (IL7R)**	8.517	23,6	12.337	19,1

C pbmc10k

	PBMC	
	# of cells	% of cells
**Detected cells**	11.485	100,0
**Cells after QC**	9.223	80,3
**CD14+ | CD11b+**	3.235	28,2
**MPO- | CD123- | CD1C-**	2.890	25,2
**APOE+|MRC1+**		
**CD68+ or CD163+**	2.339	20,4
**T cells (CD8A)**	1.516	13,2
**T cells (IL7R)**	3.997	34,8

SUPPLEMENTARY TABLE 10 Most frequent CD68/CD163 cluster-defining genes of Dataset 1 and 2.
Dataset 1: (1), Dataset 2: (2).

Gene	Data set	Cluster	Gene number	Gene	Data set	Cluster	Gene number	Gene	Data set	Cluster	Gene number	Gene	Data set	Cluster	Gene number
CCL18	1	0	1	IL1B	1	4	1	CCL18	2	0	1	CXCL8	2	4	1
MMP12	1	0	2	CXCL8	1	4	2	APOE	2	0	2	IL1B	2	4	2
C1QB	1	0	3	CXCL3	1	4	3	C1QB	2	0	3	CCL20	2	4	3
C1QA	1	0	4	CCL20	1	4	4	SLC40A1	2	0	4	G0S2	2	4	4
RNASE1	1	0	5	TIMP1	1	4	5	C1QA	2	0	5	CXCL3	2	4	5
SLC40A1	1	0	6	CXCL1	1	4	6	CTSC	2	0	6	CCL3	2	4	6
APOE	1	0	7	CXCL2	1	4	7	APOC1	2	0	7	CCL3L1	2	4	7
C1QC	1	0	8	G0S2	1	4	8	LGMN	2	0	8	C15orf48	2	4	8
CXCL9	1	0	9	CCL3	1	4	9	HLA-DQA1	2	0	9	TIMP1	2	4	9
HLA-DQA1	1	0	10	IL1RN	1	4	10	ACP5	2	0	10	PTGS2	2	4	10
HLA-DRB5	1	1	1	FN1	1	5	1	FN1	2	1	1	GZMB	2	5	1
C1QC	1	1	2	SPP1	1	5	2	FBP1	2	1	2	IL32	2	5	2
CXCL10	1	1	3	FBP1	1	5	3	SPP1	2	1	3	LTB	2	5	3
HLA-DPB1	1	1	4	RGCC	1	5	4	MARCO	2	1	4	TRAC	2	5	4
C1QA	1	1	5	MARCO	1	5	5	VIM	2	1	5	CD3D	2	5	5
C1QB	1	1	6	CSTB	1	5	6	RGCC	2	1	6	CD7	2	5	6
HLA-DQA2	1	1	7	VIM	1	5	7	CSTB	2	1	7	CD2	2	5	7
HLA-DPA1	1	1	8	S100A10	1	5	8	CHI3L1	2	1	8	CST7	2	5	8
FAM26F	1	1	9	CHI3L1	1	5	9	S100A10	2	1	9	CCL5	2	5	9
CST3	1	1	10	MT1G	1	5	10	FABP5	2	1	10	TNFRSF18	2	5	10
APOBEC3A	1	2	1	CCL5	1	6	1	CXCL10	2	2	1	RNASE1	2	6	1
FCN1	1	2	2	IL32	1	6	2	HLA-DRB5	2	2	2	SPP1	2	6	2
IFITM3	1	2	3	GNLY	1	6	3	HLA-DQA2	2	2	3	IFI27	2	6	3
IFITM2	1	2	4	TRAC	1	6	4	C1QC	2	2	4	HLA-DRB5	2	6	4
SERPINB9	1	2	5	NKG7	1	6	5	CST3	2	2	5	VMO1	2	6	5
ACTB	1	2	6	TRBC2	1	6	6	CALHM6	2	2	6	MSR1	2	6	6
RP11-1143G9.4	1	2	7	LTB	1	6	7	CXCL9	2	2	7	CD9	2	6	7
IFI30	1	2	8	GZMB	1	6	8	HLA-DPA1	2	2	8	C1QB	2	6	8
ZFP36	1	2	9	CD2	1	6	9	HLA-DPB1	2	2	9	C1QC	2	6	9
IFITM1	1	2	10	CD3D	1	6	10	C1QB	2	2	10	C1QA	2	6	10
FABP5	1	3	1	S100A9	1	7	1	S100A8	2	3	1	TXN	2	7	1
SPP1	1	3	2	S100A8	1	7	2	S100A9	2	3	2	FTL	2	7	2
SDS	1	3	3	S100A12	1	7	3	CCL2	2	3	3	SDS	2	7	3
IFI6	1	3	4	FCN1	1	7	4	TIMP1	2	3	4	IFI30	2	7	4
ALOX5AP	1	3	5	VCAN	1	7	5	APOBEC3A	2	3	5	FTH1	2	7	5
APOC1	1	3	6	TKT	1	7	6	FCN1	2	3	6	ATOX1	2	7	6
APOE	1	3	7	CSTA	1	7	7	ISG15	2	3	7	GPX4	2	7	7
CTSD	1	3	8	RPL7	1	7	8	S100A12	2	3	8	ELOB	2	7	8
NUPR1	1	3	9	THBS1	1	7	9	VCAN	2	3	9	CYP27A1	2	7	9
GPNMB	1	3	10	AP1S2	1	7	10	IFITM2	2	3	10	SOD2	2	7	10

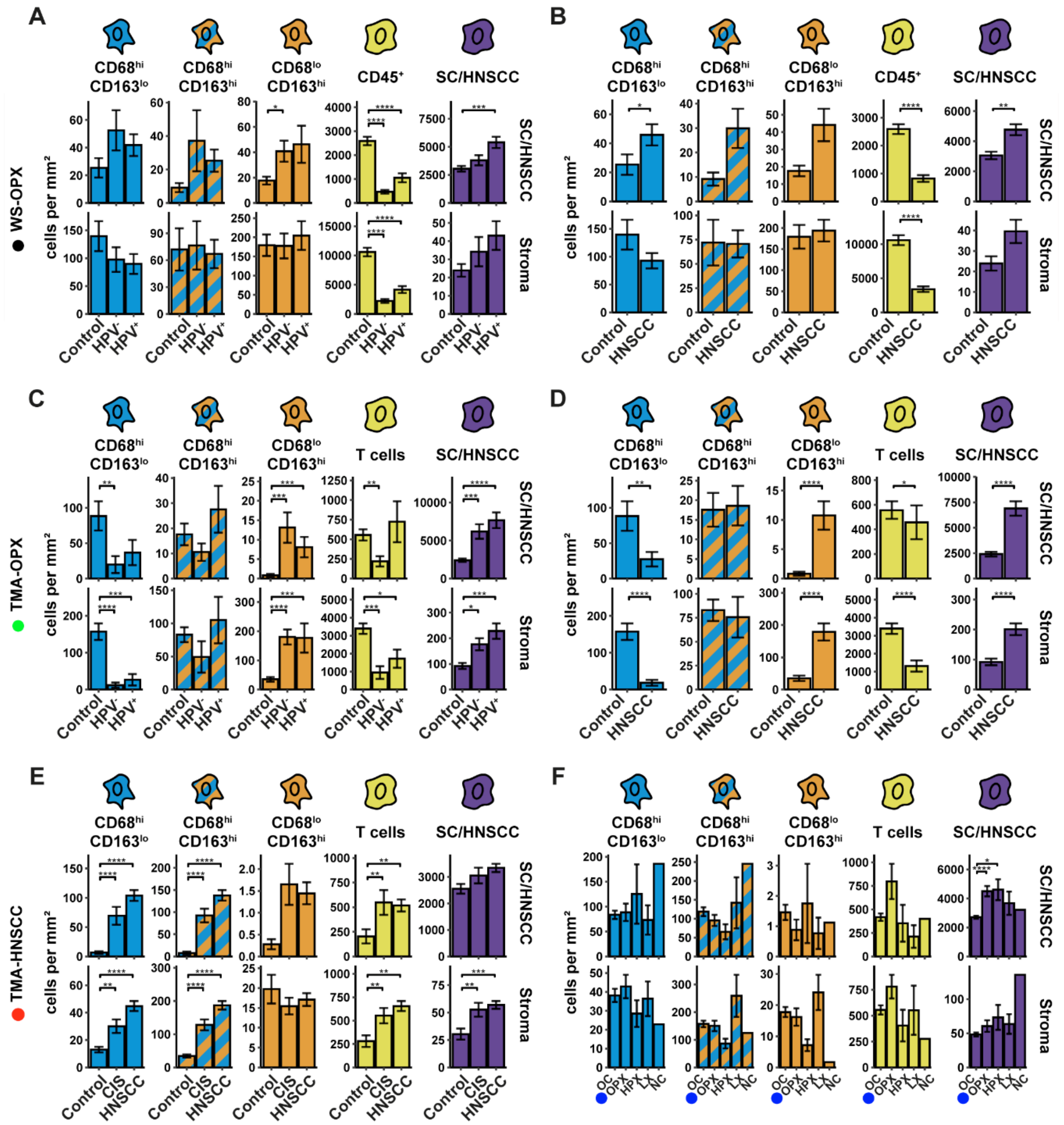

SUPPLEMENTARY FIGURE 1 Comparison of cell subset densities between whole slides and TMA data sets. **(A – F)** Quantification of cell densities (per mm^2) of the CD68/CD163 MΦ subsets, T cells ($CD3^+$) vs leukocytes ($CD45^+$), and SC/HNSCC in tumor nests (HNSCC) and stroma in controls, CIS and HNSCC. **(A)** Whole slide dataset of oropharyngeal carcinomas and tonsils of tumor-free patients as controls (WS-OPX) as shown in **Figure 1E**. **(B)** Same data as **(A)** without differentiation between $HPV^{+/-}$. HNSCCs. **(C)** 2nd TMA dataset of oropharyngeal carcinomas and tonsils of tumor-free patients as controls (TMA-OPX) with differentiation between $HPV^{+/-}$. **(D)** Same as **(C)** without differentiation between $HPV^{+/-}$. **(E)** TMA dataset of HNSCCs and corresponding CIS as well as tumor-free mucosa from tumor patients as controls (TMA-HNSCC) as shown in **Figure 3D**. **(F)** Subdivision of HNSCC TMA dataset into oral carcinomas (OC), oropharyngeal carcinomas (OPX), hypopharyngeal carcinomas (HPX), laryngeal carcinomas (LX) and nasal carcinomas (NC). For better illustration, cell subsets and datasets color-coded. The significance levels (by Wilcoxon rank sum test) between the groups are indicated as asterisks above the plots. * $p<0.05$, ** $p<0.01$, *** $p<0.001$, **** $p<0.0001$.

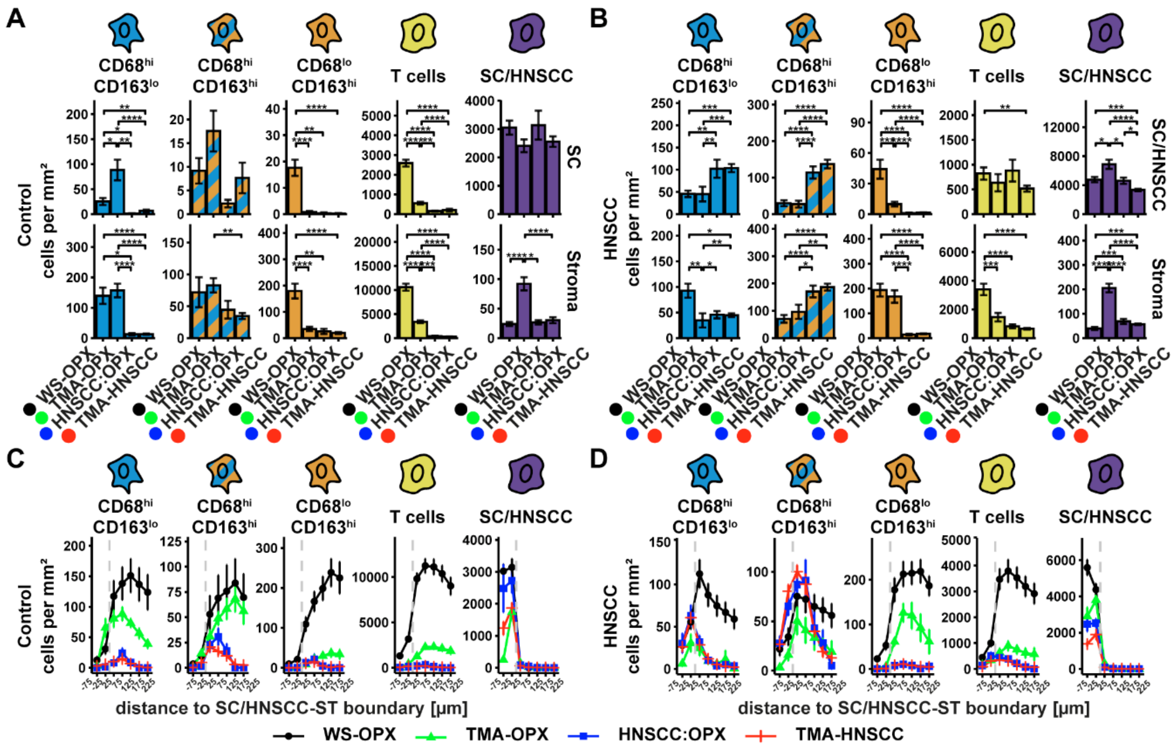

SUPPLEMENTARY FIGURE 2 Comparison of cell subset densities between whole slides and TMA data sets. Quantification of cell densities (per mm^2) of the CD68/CD163 subsets, T cells (CD3$^+$) vs leukocytes (CD45$^+$), and SC/HNSCC within control tissue **(A)** and tumor tissue **(B)** of oropharyngeal carcinomas whole slides (WS-OPX), a TMA dataset of oropharyngeal carcinomas (TMA-OPX) a selection of oropharyngeal carcinomas within our HNSCC TMA dataset (HNSCC:OPX) and all HNSCC within our HNSCC TMA dataset (TMA-HNSCC). For better illustration, cell subsets and datasets have been color-coded. The significance levels (by Wilcoxon rank sum test) between the groups are indicated as asterisks above the plots. * p<0.05, ** p<0.01, *** p<0.001, **** p<0.0001. **(C)** and **(D)** Same samples as in **(A)** and **(B)** but quantification of cell density as a function of distance to squamous epithelium (SC) or HNSCC-stroma (ST) boundary (SC/HNSCC-ST boundary). The distance to the SC/HNSCC-ST boundary is plotted on the X-axis. The vertical dashed line indicates the border between the stroma and the tumor or squamous epithelium. Negative X values reflect distances to SC/HNSCC-ST boundary within the tumors or squamous epithelium, positive X values distances in the adjacent stroma. Each line represents a group (WS-OPX = black, TMA-OPX = green, HNSCC:OPX = blue, TMA-HNSCC = red), each point represents the mean value of multiple samples. The error bars represent the standard error.

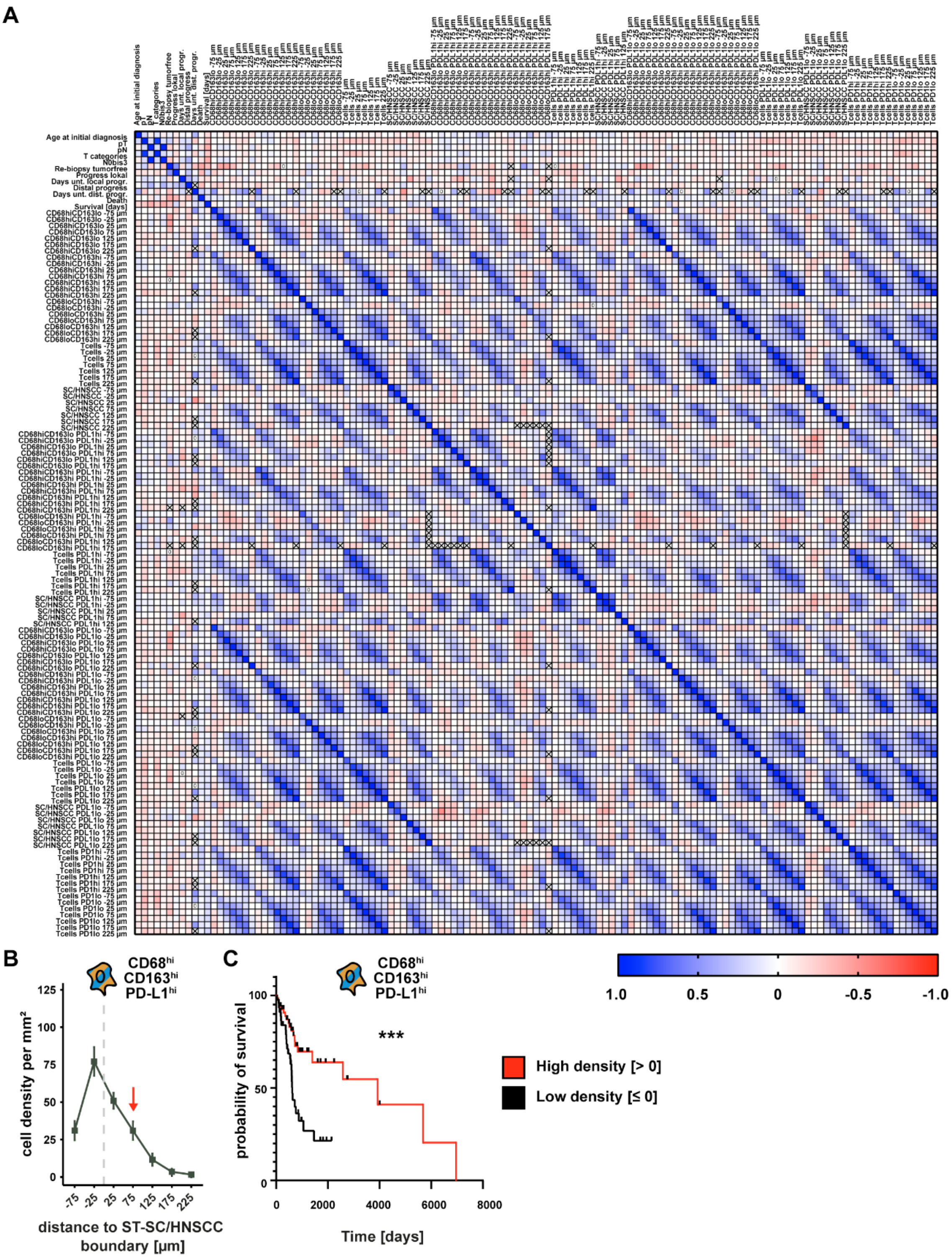

SUPPLEMENTARY FIGURE 3 Comparison of spatial cell-cell relationships with clinical outcome. **(A)** Correlation matrix (by Spearman's correlation) as a heat map of clinical events with cell density as a function of distance to squamous epithelium (SC) or HNSCC-stroma (ST) boundary (SC/HNSCC-ST boundary). This figure is a supplement to **Figure 8A**, where all correlations to the density at distance to SC/HNSCC-ST boundary are listed. The corresponding statistical data can be taken from **Supplementary Table 8**. **(B)** Cell density of CD68^{hi}CD163^{hi}PD-L1^{hi} cells as a function of distance to ST-CC/HNSCC boundary. Each point represents the mean value of multiple samples. Negative X values reflect distances to SC/HNSCC-ST boundary within the tumours or squamous epithelium, positive X values distances in the adjacent stroma. The vertical dashed line indicates the border between the stroma and the tumour or squamous epithelium. **(C)** Kaplan-Meier curves of patient survival with a high (> 0) compared to a low (≤ 0) density of CD68^{hi}CD163^{hi}PD-L1^{hi} cells at 75 µm from the ST-CC/HNSCC boundary. This curve is a complement of **Figure 8D**, with 0 chosen as the threshold here instead of the median.

The significance levels (by Log-rank (Mantel-Cox) test) between the groups are indicated as asterisks above the plots. * p<0.05, ** p<0.01, *** p<0.001, **** p<0.0001.

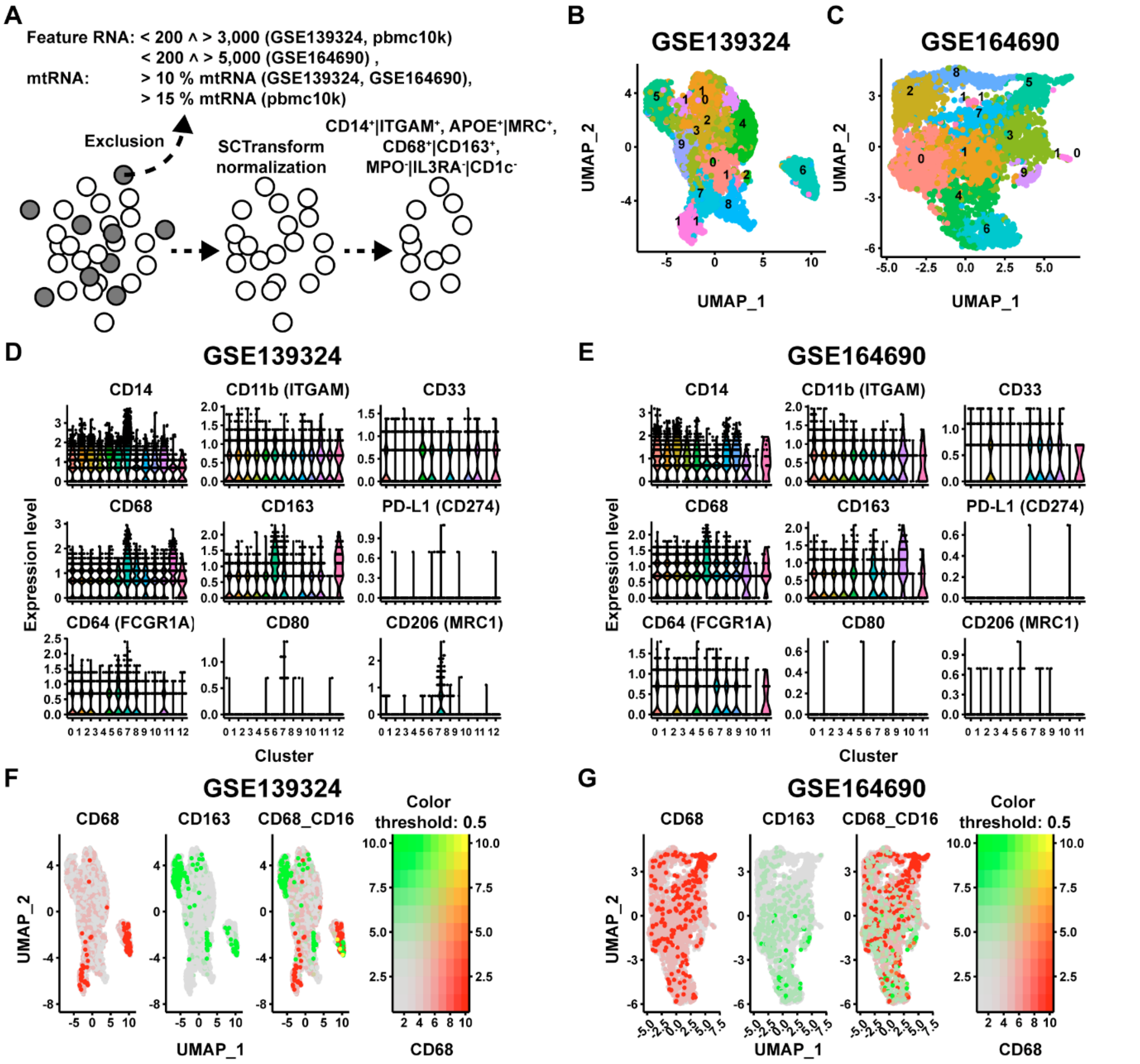

SUPPLEMENTARY FIGURE 4 Single cell NGS analysis of CD68/CD163 clusters in datasets (GSE139324 and GSE164690) of human blood cells of HNSCC patients. **(A)** Quality control (QC), metrics and selection of $CD14^{+}|CD11b^{+}$, $MPO^{-}|CD123^{-}|CD1C^{-}$, cells before further analysis. **(B, C)** UMAP visualization of the CD68/CD163 MΦ blood cell clusters of HNSCC patients (study A (GSE139324): 6736 cells, study B (GSE164690): 4839 cells). **(D, E)** Violin plots of the expression levels of CD14, CD11c, CD33, CD68, CD163, PD-L1, CD64, CD80 and CD206 within the clusters of **(A)** and **(B)**. **(F, G)** Color-coded feature plot of CD68 and CD163 expression per cell projected onto the UMAP visualization of A (GSE139324) and B (GSE164690).

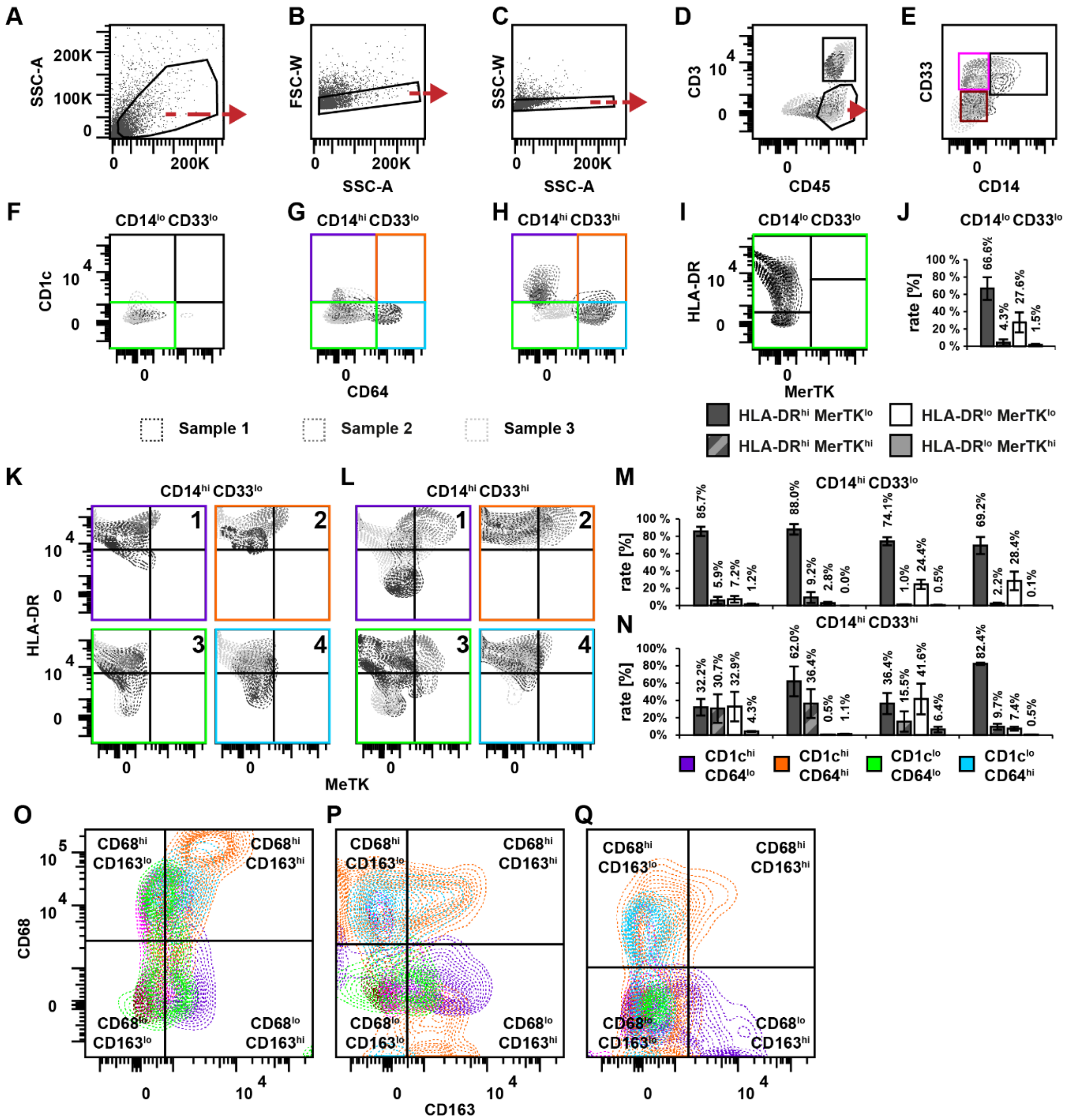

SUPPLEMENTARY FIGURE 5 Flow cytometric characterization of CD68/CD163 subsets in HNSCC. Single cell suspensions of 3 primary human HNSCCs were labelled using fluorochrome-conjugated antibody panel against CD1c, CD3, CD14, CD33, CD45, CD64, CD68, CD80, CD163, CD206, HLA-DR, MerTK and PD-L1 and measured by flow cytometer. **(A)** Leukocytes were pre-selected with forward and side scatter light. **(B – D)** After exclusion of cell duplicates, T cells were excluded as CD45^{+}CD3^{+}. **(E)** Subdivision of CD45^{+}CD3^{-} leukocytes via CD14 and CD33 into CD14^{lo}CD33^{lo} (granulocytes and B cells) CD14^{lo}CD33^{hi} (non-classical monocytes, MΦ and DCs) and CD14^{hi}CD33^{hi} cells (classical monocytes, MΦ and DCs). CD33 is strongly expressed by most monocytes, MΦ and DCs and weaklier by granulocytes and NK cells (3–7). CD14 is expressed highly on classical and intermediate monocytes, DCs, MΦ. Neutrophil granulocytes, non-classical monocytes, some DCs and MΦ do not or only weakly express CD14. **(F – H)** Differentiation of CD14/CD33 subsets via CD1c and CD64. CD1c belongs to the MHC-like CD1 genes and is involved in the presentation of lipid-based antigens to T cells (8). It is mainly expressed by DCs and to a lower extend by monocytes and B cells (8, 9). CD64 (Fc γ receptor I) is expressed by most monocytes, MΦ, neutrophil granulocytes and some DCs (10, 11). **(I – N)** Subdivision of Differentiation of CD14/CD33 subsets by HLA-DR and MerTK. The CD14^{lo}CD33^{hi} and CD14^{hi}CD33^{hi} cells here additionally subdivided into CD1c^{hi}CD64^{lo}, CD1c^{hi}CD64^{hi}, CD1c^{lo}CD64^{lo} and CD1c^{lo}CD64^{hi}. HLA-DR is expressed on a high level by MΦ, DCs, monocytes and B cells and to a lower extend by granulocytes (3, 6, 12–14). Myeloid-epithelial-reproductive tyrosine kinase (MerTK) is physiologically expressed by a variety of cell types, particularly on mature MΦ, but also on monocytes, DCs, and other hematopoietic cell types. On MΦ and DCs it is involved in the regulation of tissue homeostasis and repair as well as innate immune control via efferocytosis (15). Based on the above expression patterns, 6 leukocyte populations were distinguished for further investigation: CD14^{lo}CD33^{lo} (granulocytes and B cells), CD14^{lo}CD33^{hi}

(non-classical monocytes, MΦ and DCs), CD1c^{hi}CD64^{lo} (DC-enriched cells 1, DEC1), (CD14^{hi}CD33^{hi})CD1c^{hi}CD64^{hi} (DC-enriched cells 2, DEC2), CD1^{lo}CD64^{lo} (MΦ-enriched cells 1, MEC1), and (CD14^{hi}CD33^{hi})CD1^{lo}CD64^{hi} (MΦ-enriched cells 2, MEC2). **(O – Q)** Proportion of the leukocyte populations within the CD68/CD163 clusters separately displayed for each sample (**P** is identical with **Figure 10B**). Within bar plots each bar represents the mean value of three samples. The error bars show the standard error.

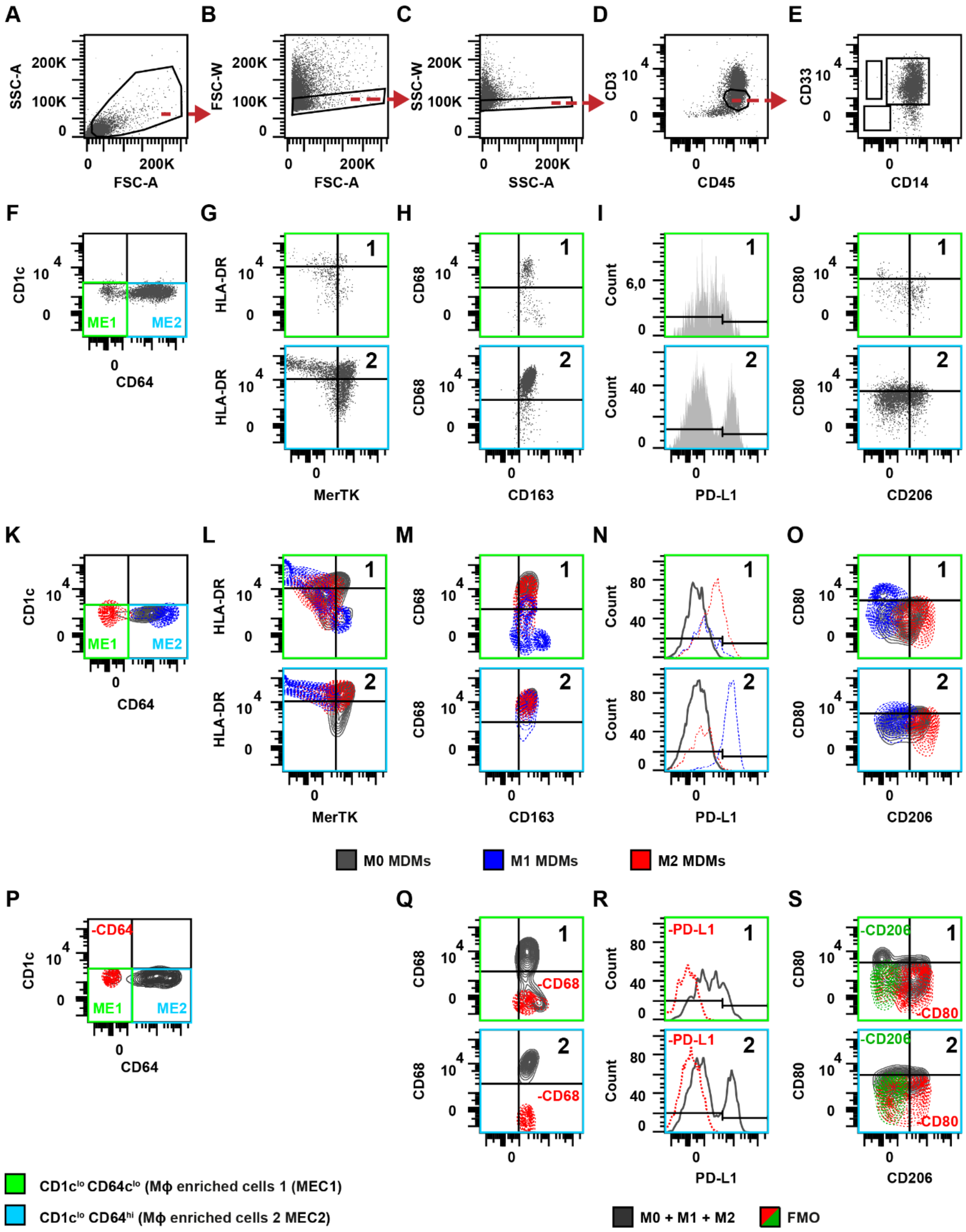

SUPPLEMENTARY FIGURE 6 Flow cytometric analysis monocyte derived MΦ (MDM). **(A – E)** Single cell suspensions of mixed M0, M1 and M2 differentiated MDMs of 2 healthy donors were labelled with the fluorochrome-conjugated antibody panel, measured by flow cytometer and gated as in **Supplementary Figure 5A – E**. **(F – J)** MEC1 and MEC2 within the CD14^{hi}CD33^{hi} cells **(F)** (mixed M0, M1 and M2) MDMs were analysed for their HLA-

DR/MerTK **(G)**, CD68/CD163 **(H)**, PD-L1 **(I)** and CD80/CD206 expression **(J)**. The gating was done as shown in **Supplementary Figure 5**. **(K – O)** MEC1 and MEC2 within the $CD14^{hi}CD33^{hi}$ cells **(K)** of M0, M1 and M2 MDMs (displayed as overlay) were analysed for their HLA-DR/MerTK **(L)**, CD68/CD163 **(M)**, PD-L1 **(N)** and CD80/CD206 expression **(O)**. Gating was performed as described above. **(P – S)** Overlay of full staining and fluorescence Minus One (FMO) controls for CD64, CD68, PD-L1 and CD206 on MDMs (mix of M0, M1 and M2 MΦ). Antibody labelling and gating was performed as described above, omitting one of the antibodies mentioned in each case.

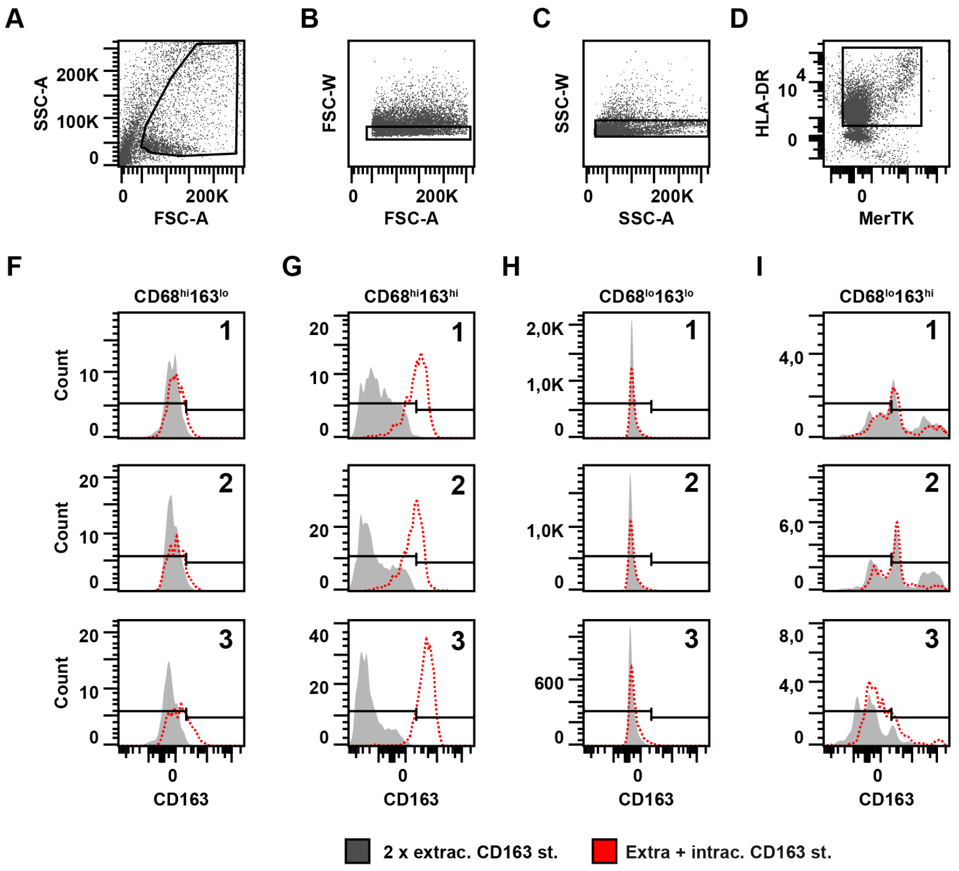

SUPPLEMENTARY FIGURE 7 Flow cytometric analysis of intracellular CD163 expression within monocyte derived MΦ (MDMs). A mix of M0, M1 and M2 polarised MDMs of 3 healthy donors were extracellularly labelled using fluorochrome-conjugated antibodies against HLA-DR, MerTK and CD163 (BV421). CD163 was used in saturating concentration. After cell permeabilization, intracellular staining was performed using CD68 and a differentially labelled CD163 (BUV496) antibody (of the same clone). In a comparison group, labelling with the second CD163 antibody was carried out before cell permeabilization and intracellular CD68 staining. Cell suspensions were then analysed by flow cytometry. **(A – E)** Leukocytes were pre-selected by forward and side scatter light **(A)** and cell duplicates were excluded **(B, C)**. Cells were further differentiated by HLA-DR and MerTK **(D)**. $HLA\text{-}DR^{+}$ cells were separated into CD68/CD163 subsets **(E)**. **(F – I)** Subsequently, for the CD68/CD163 MΦ subsets, the expression of the second CD68 antibody was shown as an overlay histogram for both intracellular staining (red) and control (grey). Each line **(F - I)** represents the CD68/CD163 subsets one donor. Each column within **(F – I)** shows 3 independent experiments (1 - 3).

References

1. Cillo, A. R., Kürten, C. H. L., Tabib, T., Qi, Z., Onkar, S., Wang, T. et al. Immune Landscape of Viral- and Carcinogen-Driven Head and Neck Cancer. *Immunity* (2020). 52:183-199.e9. doi: 10.1016/j.immuni.2019.11.014.

2. Kürten, C. H. L., Kulkarni, A., Cillo, A. R., Santos, P. M., Roble, A. K., Onkar, S. et al. Investigating immune and non-immune cell interactions in head and neck tumors by single-cell RNA sequencing. *Nat Commun* (2021). 12:7338. doi: 10.1038/s41467-021-27619-4.

3. Gorczyca, W., Sun, Z.-Y., Cronin, W., Li, X., Mau, S., Tugulea, S. Immunophenotypic pattern of myeloid populations by flow cytometry analysis. *Methods Cell Biol* (2011). 103:221–66. doi: 10.1016/B978-0-12-385493-3.00010-3.

4. Wong, K. L., Yeap, W. H., Tai, J. J. Y., Ong, S. M., Dang, T. M., Wong, S. C. The three human monocyte subsets: implications for health and disease. *Immunol Res* (2012). 53:41–57. doi: 10.1007/s12026-012-8297-3.

5. Tacke, F., Randolph, G. J. Migratory fate and differentiation of blood monocyte subsets. *Immunobiology* (2006). 211:609–18. doi: 10.1016/j.imbio.2006.05.025.

6. Ożańska, A., Szymczak, D., Rybka, J. Pattern of human monocyte subpopulations in health and disease. *Scand J Immunol* (2020). 92:e12883. doi: 10.1111/sji.12883.

7. Hejazi, M., Zhang, C., Bennstein, S. B., Balz, V., Reusing, S. B., Quadflieg, M. et al. CD33 Delineates Two Functionally Distinct NK Cell Populations Divergent in Cytokine Production and Antibody-Mediated Cellular Cytotoxicity. *Front Immunol* (2021). 12:798087. doi: 10.3389/fimmu.2021.798087.

8. Heger, L., Hofer, T. P., Bigley, V., Vries, I. J. M. de, Dalod, M., Dudziak, D. et al. Subsets of CD1c+ DCs: Dendritic Cell Versus Monocyte Lineage. *Front Immunol* (2020). 11:559166. doi: 10.3389/fimmu.2020.559166.

9. Ziegler-Heitbrock, L., Ancuta, P., Crowe, S., Dalod, M., Grau, V., Hart, D. N. et al. Nomenclature of monocytes and dendritic cells in blood. *Blood* (2010). 116:e74-80. doi: 10.1182/blood-2010-02-258558.

10. Akinrinmade, O. A., Chetty, S., Daramola, A. K., Islam, M.-U., Thepen, T., Barth, S. CD64: An Attractive Immunotherapeutic Target for M1-type Macrophage Mediated Chronic Inflammatory Diseases. *Biomedicines* (2017). 5. doi: 10.3390/biomedicines5030056.

11. Grage-Griebenow, E., Zawatzky, R., Kahlert, H., Brade, L., Flad, H., Ernst, M. Identification of a novel dendritic cell-like subset of CD64(+) / CD16(+) blood monocytes. *Eur J Immunol* (2001). 31:48–56. doi: 10.1002/1521-4141(200101)31:1<48::aid-immu48>3.0.co;2-5.

12. Kim, O. Y., Monsel, A., Bertrand, M., Coriat, P., Cavaillon, J.-M., Adib-Conquy, M. Differential down-regulation of HLA-DR on monocyte subpopulations during systemic inflammation. *Crit Care* (2010). 14:R61. doi: 10.1186/cc8959.

13. Döring, M., Cabanillas Stanchi, K. M., Haufe, S., Erbacher, A., Bader, P., Handgretinger, R. et al. Patterns of monocyte subpopulations and their surface expression of HLA-DR during adverse events after hematopoietic stem cell transplantation. *Ann Hematol* (2015). 94:825–36. doi: 10.1007/s00277-014-2287-6.

14. Katikaneni, D. S., Jin, L. B cell MHC class II signaling: A story of life and death. *Hum Immunol* (2019). 80:37–43. doi: 10.1016/j.humimm.2018.04.013.

15. Huelse, J. M., Fridlyand, D. M., Earp, S., DeRyckere, D., Graham, D. K. MERTK in cancer therapy: Targeting the receptor tyrosine kinase in tumor cells and the immune system. *Pharmacol Ther* (2020). 213:107577. doi: 10.1016/j.pharmthera.2020.107577.
